# Stress Responses of the Industrial Workhorse *Bacillus licheniformis* to Osmotic Challenges

**DOI:** 10.1371/journal.pone.0080956

**Published:** 2013-11-15

**Authors:** Rebecca Schroeter, Tamara Hoffmann, Birgit Voigt, Hanna Meyer, Monika Bleisteiner, Jan Muntel, Britta Jürgen, Dirk Albrecht, Dörte Becher, Michael Lalk, Stefan Evers, Johannes Bongaerts, Karl-Heinz Maurer, Harald Putzer, Michael Hecker, Thomas Schweder, Erhard Bremer

**Affiliations:** 1 Pharmaceutical Biotechnology, Institute of Pharmacy, University of Greifswald, Greifswald, Germany; 2 Laboratory for Microbiology, Department of Biology, University Marburg, Marburg, Germany; 3 LOEWE-Center for Synthetic Microbiology, University Marburg, Marburg, Germany; 4 Institute for Microbiology, University of Greifswald, Greifswald, Germany; 5 Institute of Biochemistry, University of Greifswald, Greifswald, Germany; 6 Henkel AG & Co. KGaA, Düsseldorf, Germany; 7 AB Enzymes GmbH, Darmstadt, Germany; 8 CNRS UPR 9073 (Affiliated with Université Paris Diderot, Sorbonne Paris Cité), Institute de Biologie Physico-Chimique, Paris, France; University of Oklahoma Health Sciences Center, United States of America

## Abstract

The Gram-positive endospore-forming bacterium *Bacillus licheniformis* can be found widely in nature and it is exploited in industrial processes for the manufacturing of antibiotics, specialty chemicals, and enzymes. Both in its varied natural habitats and in industrial settings, *B. licheniformis* cells will be exposed to increases in the external osmolarity, conditions that trigger water efflux, impair turgor, cause the cessation of growth, and negatively affect the productivity of cell factories in biotechnological processes. We have taken here both systems-wide and targeted physiological approaches to unravel the core of the osmostress responses of *B. licheniformis*. Cells were suddenly subjected to an osmotic upshift of considerable magnitude (with 1 M NaCl), and their transcriptional profile was then recorded in a time-resolved fashion on a genome-wide scale. A bioinformatics cluster analysis was used to group the osmotically up-regulated genes into categories that are functionally associated with the synthesis and import of osmostress-relieving compounds (compatible solutes), the SigB-controlled general stress response, and genes whose functional annotation suggests that salt stress triggers secondary oxidative stress responses in *B. licheniformis*. The data set focusing on the transcriptional profile of *B. licheniformis* was enriched by proteomics aimed at identifying those proteins that were accumulated by the cells through increased biosynthesis in response to osmotic stress. Furthermore, these global approaches were augmented by a set of experiments that addressed the synthesis of the compatible solutes proline and glycine betaine and assessed the growth-enhancing effects of various osmoprotectants. Combined, our data provide a blueprint of the cellular adjustment processes of *B. licheniformis* to both sudden and sustained osmotic stress.

## Introduction

*Bacillus licheniformis* is a Gram-positive endospore-forming microorganism that is widely distributed in nature and can readily be isolated from soils and animal and plant material [1-5]. It is extensively exploited in industrial processes [6-8]. In particular, the excellent protein secretion capacities of *B. licheniformis* [9,10] have made it an attractive host for the large-scale production of commercially employed enzymes (e.g., amylases, phytases, proteases). It is generally regarded as safe, and since some strains of this species are considered as probiotic, *B. licheniformis* is also used in the food and feed industry [11,12], but it can also be considered as a food spoilage bacterium [5]. 

The sequencing of the genomes of two closely related *B. licheniformis* strains, *B. licheniformis* DSM 13^T^ [13] and *B. licheniformis* ATTC 14580 [14], has provided a blueprint for further in-depth studies of the physiology of this industrial workhorse and incentives for the rational design of industrial relevant strains with improved production capabilities [15-18] and enhanced biosafety [19]. Genome-wide transcriptomic and proteomic investigations of stressed *B. licheniformis* cells have allowed detailed insights into its genetic regulatory circuits, metabolic networks, biosynthetic capabilities, and cellular stress adaptation responses [10,20-26]. These studies have provided valuable knowledge when one considers that large-scale and high-density fermentation processes impose considerable constraints on microbial cells and can impair their fitness and capacity to produce biotechnologically valuable products efficiently [27-29]. 

 An important challenge with which *B. licheniformis* has to cope, both in its varied natural habitats [1-5] and in industrial settings [30], are fluctuations in the external osmolarity. In its soil ecosystem, *B. licheniformis* will be frequently exposed on one hand to high osmolarity micro-niches that are caused by desiccation processes and increases in salinity; on the other hand, rainfall and flooding of the soil habitat will confront the cell with rapid osmotic down-shifts. During biotechnological applications, high-level excretion of metabolites and substrate feed will increase the osmolarity of the growth medium [27,30,31], a process leading to water efflux from microbial cells that causes a reduction in turgor and eventually leads to cessation of growth [32]. Hence, the overall productivity of *B. licheniformis* cell factories will be negatively affected by high-osmolarity growth conditions. However, the cellular adjustment process to either suddenly imposed or sustained osmotic stress is not well studied in this *Bacillus* species [33,34].

As its cousins *Bacillus subtilis* [35,36] and *Bacillus cereus* [37], *B. licheniformis* possesses a general stress response system that is under the control of the alternative transcription factor SigB [37,38]. Members of the SigB-controlled regulon provide pre-emptive stress resistance to a multitude of environmental insults, various cellular constraints, and nutritional limitations [35-37]. Detailed studies in *B. subtilis* have shown that high salinity is one of the most-strongest inducers of the general stress response [39,40], and many members of its SigB regulon contribute to survival when the cells are exposed to severe and growth limiting osmotic up-shocks [41,42]. However, due to the transient nature of the induction of the SigB-regulon in response to acute salt stress [40,43,44], this general stress response system is not crucial for the ability of *B. subtilis* to strive under sustained high-salinity growth conditions [43]. Under these circumstances, an effective cellular water management is key to ascertaining a physiologically adequate level of hydration of the cytoplasm and magnitude of turgor in order to sustain growth [32,45]. 

Despite the existence of water-conduction channels, the aquaporins, in a considerable number of microorganisms [46], it is worth recalling that no bacterial cell can actively pump water across the semi-permeable cytoplasmic membrane to compensate for the water influxes or effluxes instigated by changes in the external osmolarity [32,47]. As a consequence, microorganisms have to balance the vital osmotic gradient across their cytoplasmic membrane indirectly by influencing the osmotic potential of the cytoplasm to direct the flux of water in or out of the cell [48,49]. They accumulate water-attracting ions and organic osmolytes (compatible solutes) when they face hyperosmotic conditions to prevent cellular dehydration [32,48], and they rapidly expel these compounds through the transient opening of mechanosensitive channels to avert cell rupture when the osmolarity suddenly drops [50-52].

The cellular stress response to high osmolarity is typically a multi-phasic process [32]. It initially entails in many microorganisms the rapid uptake of K^+^ ions as an emergency reaction [53] and the subsequent replacement of this ion with a class of organic osmolytes that are highly compliant with cellular physiology, the compatible solutes [48]. Synthesis and uptake of the compatible solutes glycine betaine and proline play a key role in the defense of *B. subtilis* against the insults of high salinity [53-64]. A qualitative assessment by natural abundance ^13^C-NMR spectroscopy has previously revealed that *B. licheniformis* belongs to the group of Bacilli that synthesize large amounts of proline when they are continuously exposed to high salinity surroundings [33,34]. 

While an effective water management is certainly the cornerstone for the cellular response to high salinity by most microorganisms [32,47], the overall adjustment process to this environmental challenge is a rather complex process. This is evident from genome-wide transcriptomic and proteomic assessments of the responses of *B. subtilis* and of the food pathogen *B. cereus* to acute and sustained high salinity environments [39,41,65,66]. Here we have combined physiological approaches, multi-omics techniques and genome mining to derive a comprehensive picture of the molecular and cellular events that allow the adaptation of *B. licheniformis* to high osmolarity surroundings. 

## Results and Discussion

### Assessment of the resistance of *B. licheniformis* against salt stress

**Figure 1:**
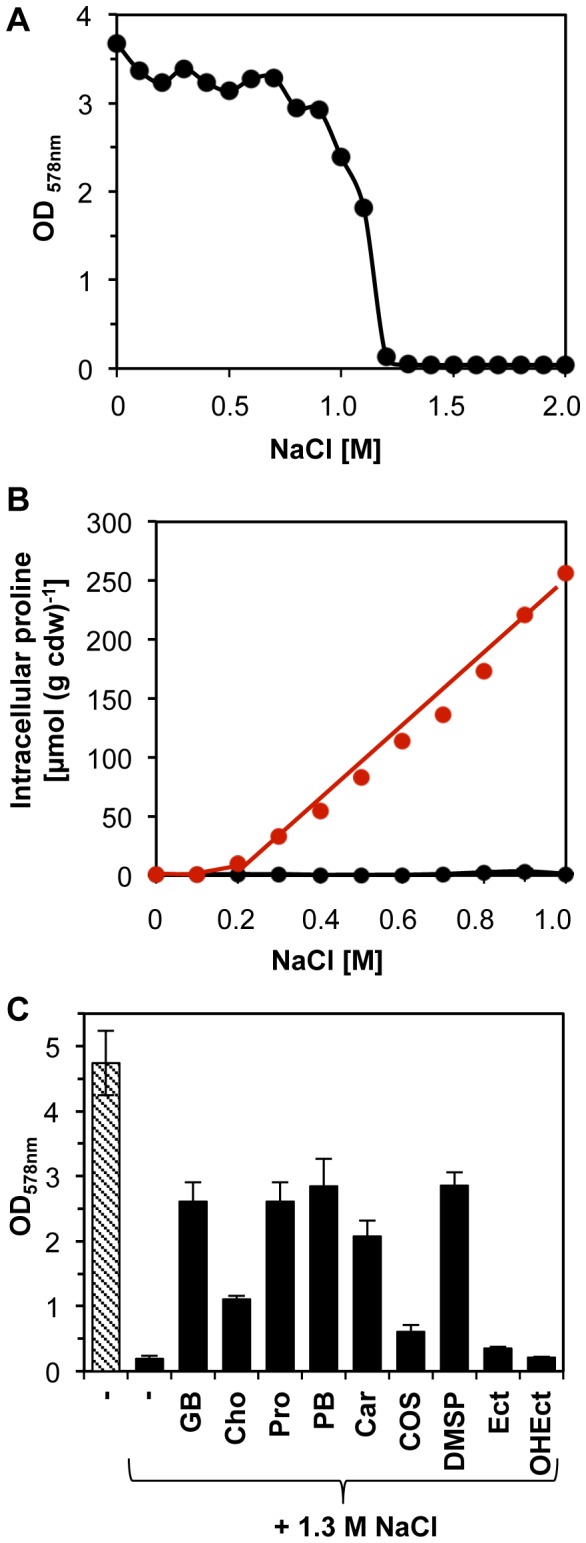
Growth yields, proline production and osmoprotection of *B. licheniformis* DSM 13^T^ by compatible solutes. (A) Cultures of *B. licheniformis* DSM13^T^ were grown at 37° C in SMM with glucose as the carbon source in the presence of the indicated NaCl concentrations. Growth yields of the cultures (as assessed by measuring the OD_578_) were determined after 14 h of incubation. (B) Proline content of osmotically stressed *B. licheniformis* DSM 13^T^ cells. Cultures were grown in SMM with the indicated salinities either in the absence (red symbol) or in the presence (black symbol) of 1 mM of the osmoprotectant glycine betaine to an optical density (OD_578_) of approximately 2. The proline content of the cells was determined by HPLC analysis. The data shown represent one typical experiment. (C) Salt-stress protection of *B. licheniformis* DSM 13^T^ by exogenously provided compatible solutes. Cultures of *B. licheniformis* DSM 13^T^ were grown in SMM either in the absence (hatched bar) or in the presence of 1.3 M NaCl (black bars) in the absence (-) or in the presence of various compatible solutes. GB: glycine betaine; Cho: choline; Pro: proline; PB: proline betaine; Car: carnitine; COS: choline-O-sulfate; DMSP: dimethylsulfoniopropionate; Ect: ectoine; OHEct: hydroxyectoine. The compatible solutes were added to the growth medium at a final concentration of 1 mM. Growth yields of the cultures were measured after 14 h of incubation at 37 °C in a shaking water bath. The data shown were derived from two independently grown cultures.

 To assess the resistance of *B. licheniformis* DSM 13^T^ against the growth-inhibiting effects of high salinity, we grew cells in a chemically defined medium (SMM) with glucose as the carbon source and different salinities in shake-flask experiments at 37° C for 14 h and then determined the growth yield of the cultures by measuring their optical densities (OD_578_). As shown in Figure 1A, B *. licheniformis* DSM 13^T^ can readily withstand salt concentrations up to 1 M NaCl but a further increase in the external salinity rapidly leads to a strong decline in growth yield; the presence of 1.3 M NaCl in the minimal medium resulted to a complete inhibition of growth. This osmotic stress resistance profile of *B. licheniformis* DSM 13^T^ is similar, but not identical, to that of *B. subtilis* [60]. *B. licheniformis* DSM 13^T^ is thus a representative of the group of Bacilli exhibiting an intermediate degree of osmotic stress resistance, and most of these species synthesize proline as their dominant osmoprotectant [33,34]. Bacilli that exhibit a considerably higher degree of salt tolerance (e.g., *Virgibacillus salexigens*) than *B. licheniformis* DSM 13^T^ or *B. subtilis* typically synthesize the compatible solute ectoine, whereas those Bacilli that synthesize only glutamate as their osmoprotectant (e.g., *B. cereus*) are rather salt-sensitive species [33,34,66]. 

### High salinity triggers a finely tuned adjustment in the cellular proline pool

 Using natural abundance ^13^C-NMR spectroscopy, it has previously been found that *B. licheniformis* DSM 13^T^ produces increased amounts of proline when cells are grown in SMM containing 1 M NaCl [34]. To investigate this salt-stress responsive *de novo* proline biosynthesis in greater detail, we grew cultures of *B. licheniformis* DSM 13^T^ to the same optical density (OD_578_ = 2) in SMM with different salinities and then measured the proline content of the cells by HPLC analysis (Figure 1B). An increase of the external salinity up to 0.2 M NaCl had little effect on the proline content of the cells, but further increases led to a graded rise in the cellular proline pool in a fashion that was directly proportional to the degree of the imposed osmotic stress (Figure 1B). Hence, it can be inferred from this experiment that *B. licheniformis* DSM 13^T^ must have the ability to detect incremental increases in the external salinity and that the cell is then able to genetically convert this information into the build-up of a situation-conform pool of an osmostress-relieving compound. The size of the proline pool at a given external salinity was strongly influenced by the presence of 1 mM glycine betaine in the growth medium. Externally provided glycine betaine is an excellent osmoprotectant for *B. licheniformis* DSM 13^T^ (Figure 1C) and its presence in the growth medium repressed the build-up of the osmostress responsive proline pool entirely (Figure 1B). Hence, osmotically challenged *B. licheniformis* DSM 13^T^ cells preferred the import of a preformed osmoprotectant such as glycine betaine over the synthesis of proline, the only compatible solute that they can produce *de novo* [33,34]. The same phenomenon has also been observed in *B. subtilis* [54,57] and it might be connected with the cell’s attempt to cope with bioenergetic constraints under high-salinity growth conditions [67] or to optimize the solvent properties of the cytoplasm [47]. 

### Osmotic stress induced changes in the transcriptome and proteome of *B. licheniformis*

**Figure 2:**
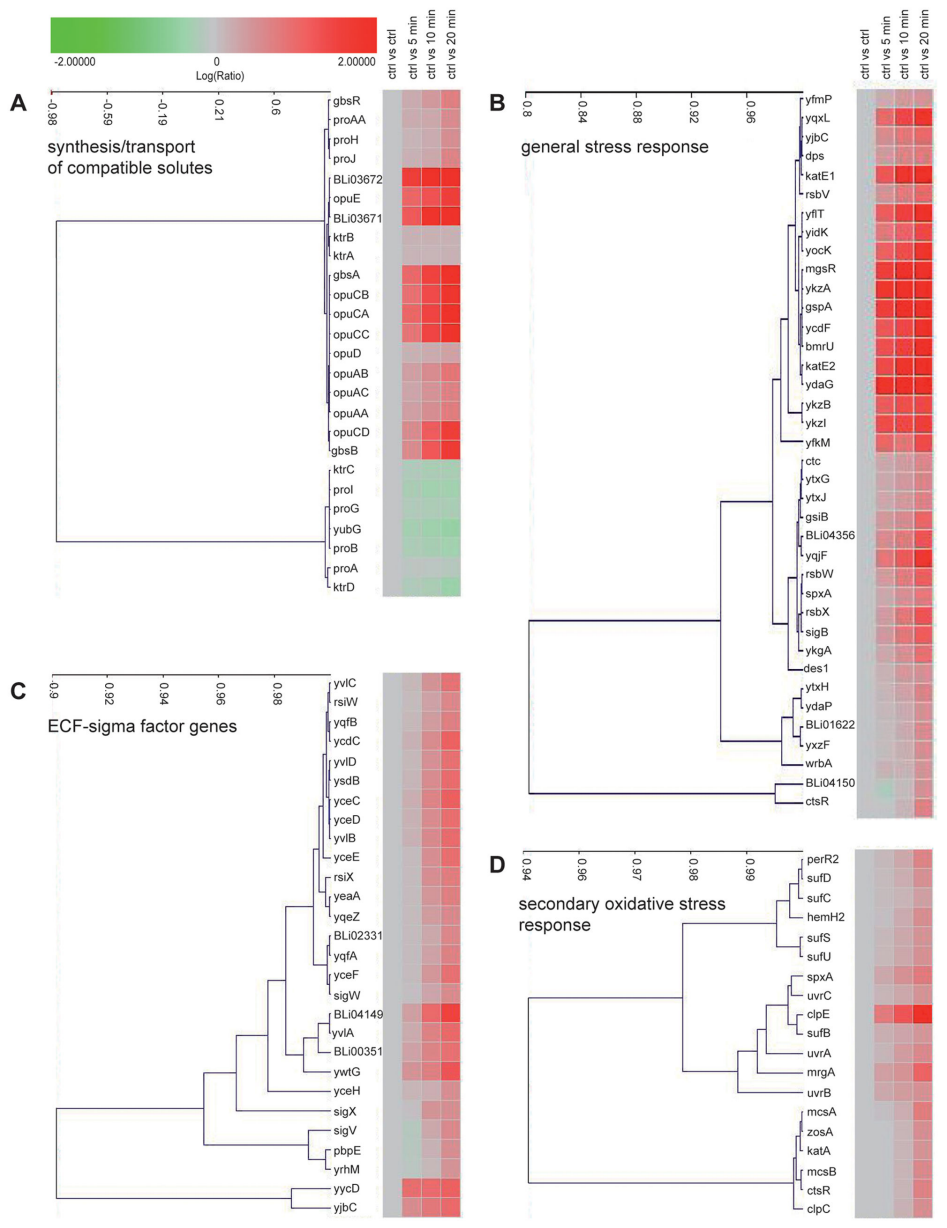
Cluster analysis of transcriptional changes in response to a sudden salt challenge. Cells of *B. licheniformis* DSM 13^T^ were cultivated in BMM with glucose as the carbon source until they reached early exponential growth phase (OD_500nm_ of about 0.4) when they were exposed to a sudden salt shock (the final NaCl concentration in the growth medium was 1.0 M). Immediately before and at the indicated time intervals subsequent to the imposed increase in the external salinity, cells were withdrawn and used for the isolation of total RNA for a genome-wide transcriptional analysis. The derived data were then subjected to a cluster analysis and grouped according to known salt stress response clusters from *B. subtilis* [39,65]: (A) Synthesis and transport of compatible solutes, (B) general stress responses, (C) ECF-sigma factor genes, and (D) secondary oxidative stress response. The correlation of the transcription patterns of the different clustered genes is represented on the X-axis (cosine correlation). Detailed values for the transcriptional profile of individual genes are given in Table S1. Genes marked in red represents those whose transcription is up-regulated in response to osmotic stress.

**Figure 3:**
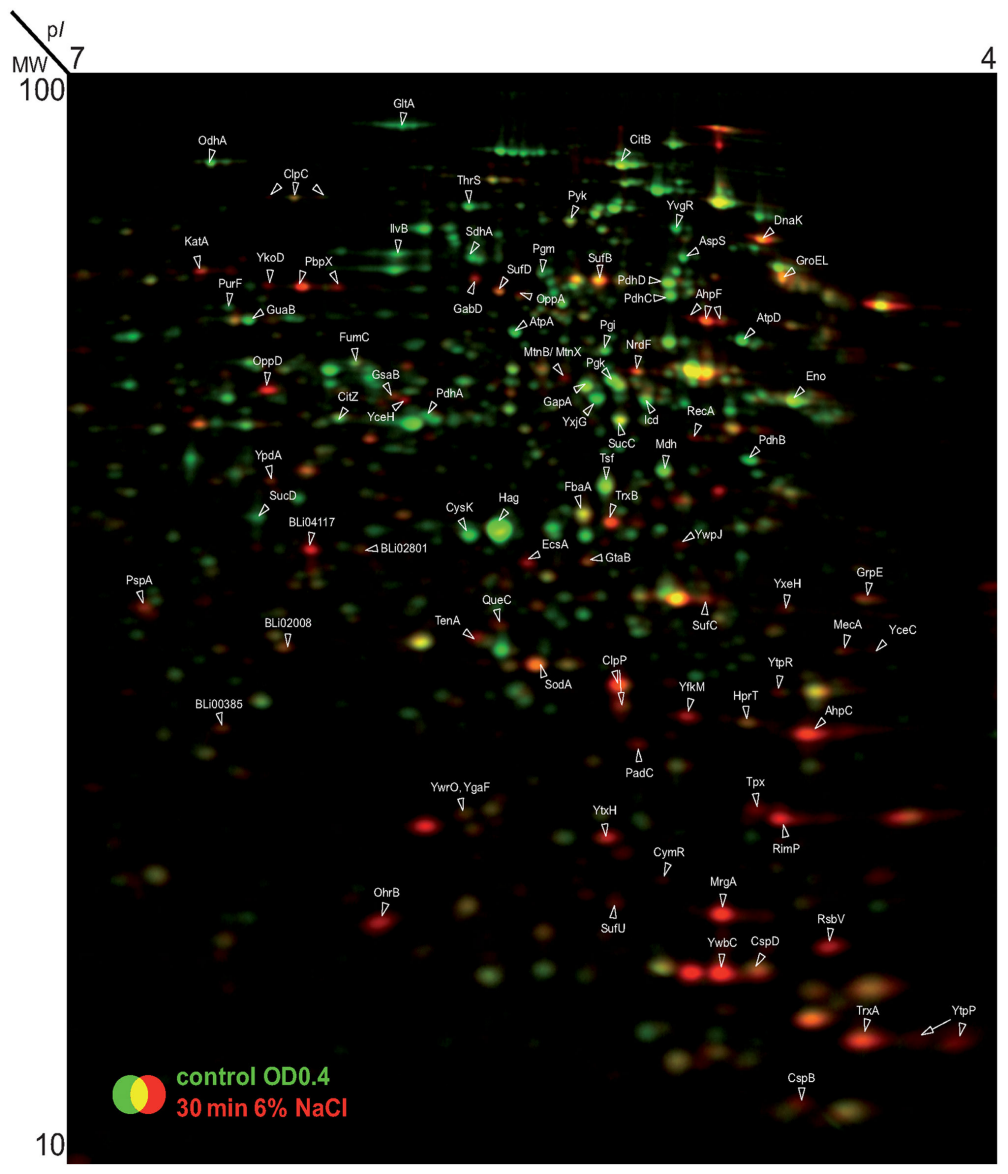
The cytosolic proteome of salt-stressed *B. licheniformis* DSM 13^T^ cells 30 min after the imposition of a NaCl shock. Cells of *B. licheniformis* DSM 13^T^ were cultivated in BMM with glucose as the carbon source until they reached early exponential growth phase (OD_500nm_ of about 0.4) when they were exposed to a sudden salt shock (the final NaCl concentration in the growth medium was 1.0 M). 30 min after the exposure to the salt stress, samples of cells were harvested and processed for 2D-PAGE analysis; proteins were separated in a pH gradient 4‑7. The control sample of the cells was harvested just prior to the imposed salt shock. Cell samples were labeled with L-[^35^S]-methionine during the exponential growth phase (control, OD_500nm_ 0.4) and 30 min after the addition of NaCl. Dual channel images were created from the 2D-gels with the Delta 2D software (Decodon GmbH, Greifswald, Germany).

 To assess the cellular response of *B. licheniformis* DSM 13^T^ cells to high salinity on a systems-wide level, we performed both a transcriptome (Figure 2) and a proteome (Figure 3) analysis with cells that were subjected to a strong osmotic up-shift (elicited with 1 M NaCl) and then analyzed the transcriptional profile time-resolved fashion (0, 5, 10, 20 min). From the 4178 *B. licheniformis* DSM 13^T^ genes covered by the DNA-array slides, we found 246 genes to be significantly up-regulated (at least 3-fold) (Table S1) and 166 genes to be significantly down-regulated (at least 3-fold) (Table S1). A summary of all genome-wide expression data collected in this study is given in Table S2. Putative regulons whose transcription was affected by high salinity were predicted from the annotation of the *B. licheniformis* DSM 13^T^ genome sequence [13] as detailed by Schroeter et al. [68]. The transcriptome data were then subjected to a cluster analysis and were binned according to known salt-stress response gene groups of *B. subtilis* [39,65]. These gene clusters comprised those that are functionally connected with: (i) the synthesis and uptake of compatible solutes, (ii) SigB-controlled general stress responses, (iii) ECF sigma factor dependent genes, and (iv) salt-stress-elicited secondary oxidative stress responses. Heat-maps representing the transcriptional profile of these four groups in *B. licheniformis* DSM 13^T^ are shown in Figure 2. 

In parallel to the analysis of the transcriptional response of *B. licheniformis* DSM 13^T^ to salt stress, we also analyzed its cellular response to this environmental cue at the level of the proteome by 2D-gel analysis (Figure 3). The synthesis of proteins subsequent to the imposition of salt stress was monitored by labeling the cells with [^35^S]-methionine and assessing the incorporation of radiolabeled methionine at time points 10 min and 30 min after an osmotic up-shock with 1M NaCl. This analysis revealed 59 proteins whose synthesis was up-regulated at least at one time point during the imposed osmotic stress (Figure 3) (Table S3). In addition, the accumulation of specific proteins at a time of 2 hours subsequent to the inflicted salt stress was determined. A higher accumulation in stressed cells compared to control cells was found for about 300 proteins (Table S4), indicating that the adjustment of *B. licheniformis* DSM 13^T^ cells to a suddenly inflicted severe salt stress is a dynamic process that extends over a considerable time period. 

### The initial salt stress response: uptake of potassium

**Figure 4:**
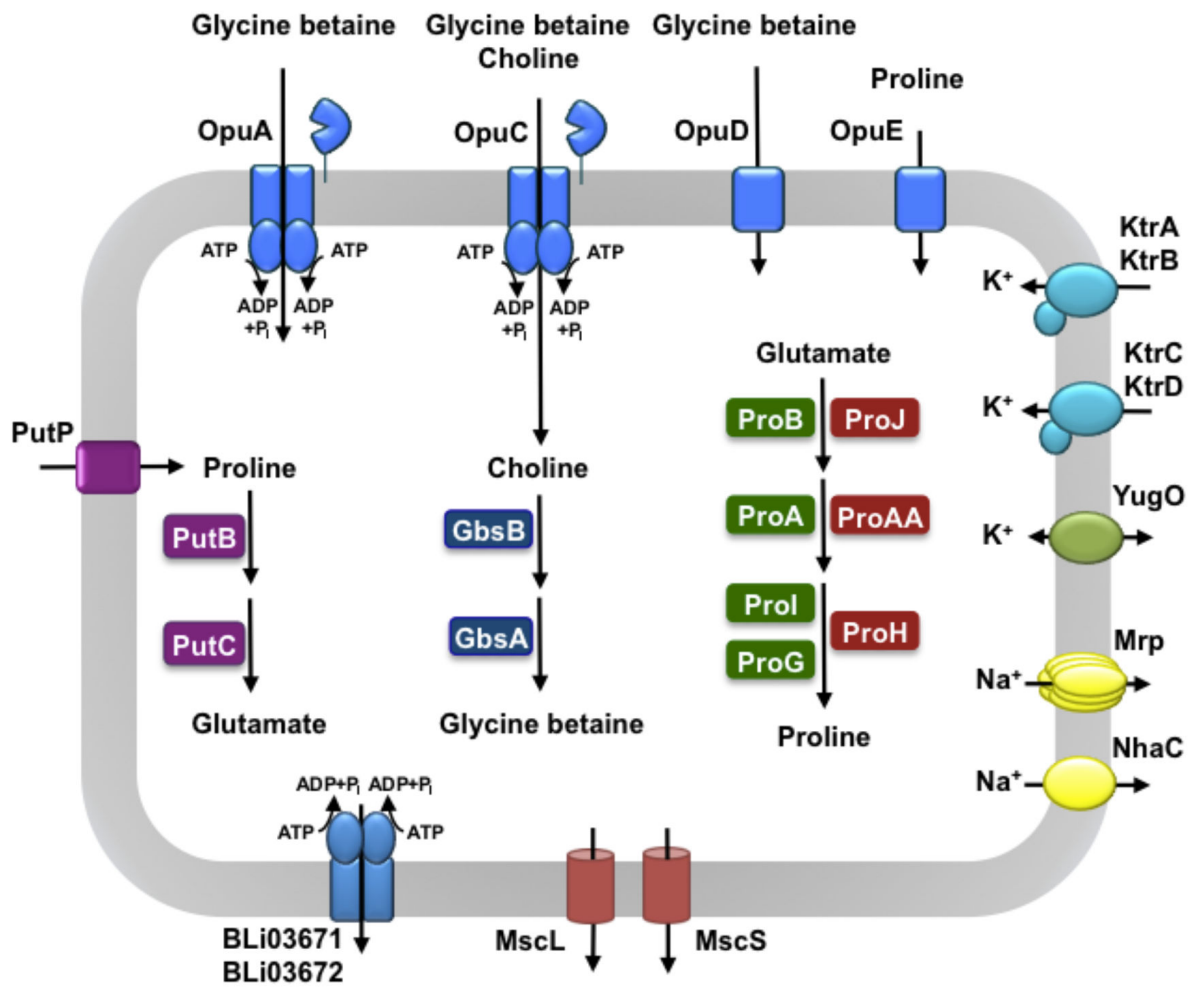
Cellular components involved in the osmostress response of *B. licheniformis* DSM 13^T^. The genome of the *B. licheniformis* DSM 13^T^ strain [13] was mined for transporters and channels that could potentially contribute to osmostress resistance. Membrane-localized proteins involved in uptake or release of osmotically relevant compounds, as well as cytoplasmic enzymes catalyzing the synthesis of the compatible solute glycine betaine and the catalytic steps for the anabolic (green enzyme symbols) and osmo-stress-adaptive (red enzyme symbols) proline biosynthetic routes are presented. These systems were identified based on their homology to their functionally characterized counterparts in *B. subtilis* [32,45,52,54,61,69,71,72]. In addition, the system responsible for the uptake (PutP) and catabolism (PutB-PutC) of externally provided L-proline [82] is shown. The physiological function of the potential exporter system Bli03671/Bli03672 [13], a member of the ABC-superfamily, whose transcription was drastically induced in response to salt stress (Table S1 and S2), is as yet unknown. MscS and MscL are mechanosensitive channels whose transient gating subsequent to an osmotic down-shock prevents cell lysis [52] since they release non-specifically water-attracting ions and organic compounds to reduce the osmotic potential of the cytoplasm and thereby curb water influx [50,51].

 The initial physiological adjustment reaction of *B. subtilis* to a sudden up-shift in the external osmolarity consists of a rapid import of potassium ions to curb the outflow of water [53,69]. Mining of the *B. licheniformis* DSM 13^T^ genome sequence [13] showed that the two potassium importers (KtrAB and KtrCD), critical for the initial stress salt stress response of *B. subtilis* [69], are also present in *B. licheniformis* DSM 13^T^ (Figure 4). However, the expression of their structural genes, as reported for *B. subtilis* [69], is not up-regulated in response to osmotic stress (Figure 2A). A third, low level, potassium import activity can be detected in *B. subtilis* strains defective in the KtrAB and KtrCD systems [69]. It might be mediated by YugO (Figure 4), a functionally poorly characterized system that is related to potassium channels and that has recently been shown to be involved in biofilm formation in *B. subtilis* [70]. There was no up-regulation in the expression of the *yugO* gene in *B. licheniformis* DSM 13^T^ (Table S2). 

 Sodium ions are highly cytotoxic and *B. subtilis* up-regulates genes encoding several exporters for this ion, including Mrp, NhaK and NhaC, when it is suddenly exposed to high NaCl concentrations [39]. *B. licheniformis* DSM 13^T^ possesses homologs to some of these sodium exporters (e.g., Mrp and NhaC) (Figure 4), but the transcription of the corresponding structural genes were not up-regulated in response to salt stress (Table S2). 

### The sustained salt stress response: uptake of osmoprotectants and synthesis of glycine betaine

 We observed a strong up-regulation in the transcription of the genes encoding transport systems (Figure 4) for compatible solutes: the expression levels of the genes for the OpuA (*opuA*-*opuAB*-*opuAC*), OpuC (*opuCA*-*opuCB*-*opuCC-opuCD*) and OpuE (*opuE*) transporters [32,45,55,62-64,71] were 3- to 90-times higher than found under control conditions (Figure 2) (Table S1 and S2). Transcription of the *opuD* gene [63] was also induced but its induction ratio did not match the criterion chosen in this study to represent a significant fold of induction (3-fold) in response to salt stress (Table S2). We tested the functionality of compatible solute import systems through osmostress protection assays [60] where we strongly impaired the growth of *B. licheniformis* DSM 13^T^ by the addition of 1.3 M NaCl to SMM and then monitored the stress-relieving effects of various types of compatible solutes on cell growth. As shown in Figure 1C, B *. licheniformis* DSM 13^T^ can derive osmoprotection from a spectrum of externally provided compatible solutes (e.g., glycine betaine, proline, carnitine), osmoprotectants that it will likely encounter in its varied soil habitats and through its association with plant and animal tissues [1-5]. The spectrum of the osmoprotectants that can be exploited by *B. licheniformis* DSM 13^T^ (Figure 1C) is similar, but not identical, to those usable by *B. subtilis* [45,71]. 

In *B. subtilis*, two very closely related osmoprotectant uptake systems, OpuB and OpuC, exist that are members of the ABC superfamily of transporters. They probably evolved through a gene duplication event [64] with a subsequent evolution of their strikingly different substrate specificities [71]. The OpuB and OpuC ABC transport systems can be distinguished, however, based on the amino acid sequence of their extracellular ligand-binding proteins (OpuBC and OpuCC) that are tethered to the cytoplasmic membrane via an N-terminally attached lipid moiety [64]. OpuBC and OpuCC are the least conserved component (69% amino acid sequence identity) of the OpuB and OpuC transporters [64]. Based upon this criterion, *B. licheniformis* DSM 13^T^ does not possess an OpuB-type transporter (Figure 4), a system that is highly specific in *B. subtilis* for the import of choline, the precursor for glycine betaine synthesis [59-61,64], whereas OpuC is an uptake system with a highly promiscuous substrate profile that also includes choline [45,71]. 

**Figure 5:**
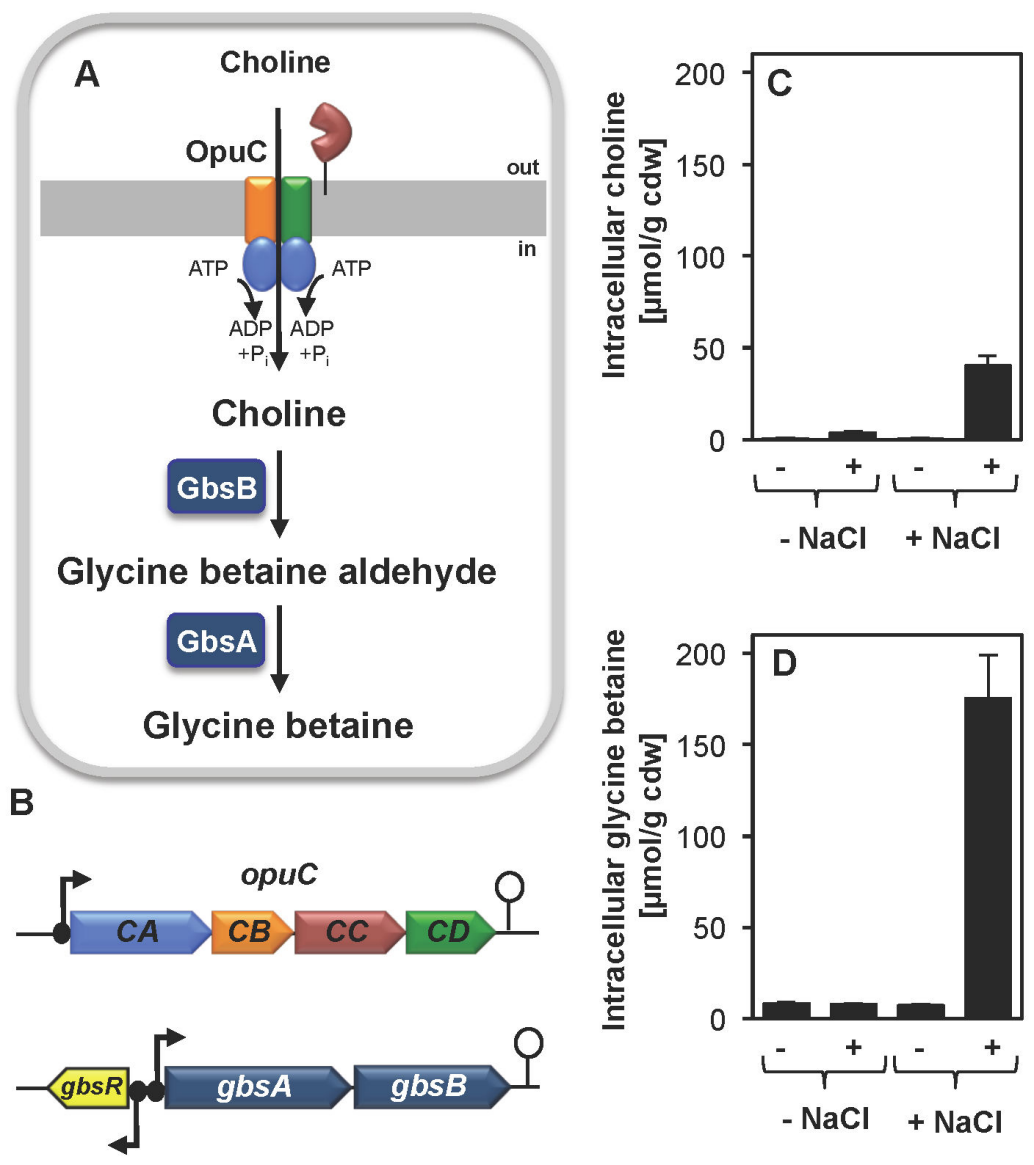
Synthesis of glycine betaine from the precursor choline by *B. licheniformis* DSM 13^T^ in response to high salinity. (A) Externally provided choline is predicted to be taken up via the ABC-transporter OpuC [64] and then converted into the compatible solute glycine betaine in a two-step oxidation reaction that involves the GbsB (choline dehydogenase) and GbsA (glycine betaine aldehyde dehydrogenase) enzymes in *B. subtilis* [61] and their counterparts in *B. licheniformis* DSM 13^T^. (B) Genetic organization of the osmotically inducible *opuC* [*opuCA-opuCB-opuCC-opuCD*] cluster encoding the OpuC transporter, the *gbsAB* biosynthetic operon, and the *gbsR* regulatory gene [59] in the genome of *B. licheniformis* DSM 13^T^ [13]. (C) The intracellular choline and (D) glycine betaine content of the cells were analyzed by ^1^H-NMR spectroscopy from unstressed and NaCl-stressed cells (the final NaCl concentration in the growth medium was 1 M). For this experiment, the cells were grown to mid-exponential growth phase in BMM or BMM with 1 M NaCl either in the absence (-) or in the presence of 1 mM choline (+ Cho). Intracellular choline and glycine betaine concentrations were absolutely quantified and normalized to cell dry weight (CDW) [nmol/mg CDW]. The error bars give the standard deviation of three independently grown cultures.

Glycine betaine accumulation via synthesis from the precursor choline is an effective osmoprotection mechanism in *B. subtilis*. Choline is imported via OpuB and OpuC and subsequently oxidized by the alcohol dehydrogenase GbsB and the glycine betaine aldehyde dehydrogenase GbsA (Figure 5A) [60,61,64].. Expression of the genes encoding the choline-specific importer OpuB and the GbsAB glycine betaine biosynthetic enzymes are controlled by the choline-responsive repressor protein GbsR [59], whose structural gene is found in a divergent orientation from the *gbsAB* operon, both in *B. subtilis* [59] and in *B. licheniformis* (Figure 5B). We found a very strong increase in the expression level of the *gbsAB* genes in *B. licheniformis* DSM 13^T^ (up to 90-fold) (Figure 2) (Table S1 and S2) in response to acute salt stress and also observed that the transcription of the *gbsR* regulatory gene was significantly induced (five-fold) (Figure 2) (Table S1 and S2) under these conditions as well. 

The strong transcriptional induction of the OpuC transport system that is expected to mediate choline import in *B. licheniformis* DSM 13^T^ and that of the GbsAB glycine betaine biosynthetic enzymes in response to high osmolarity strongly suggested that this Bacillus species should be able to synthesize glycine betaine though the oxidation of choline (Figure 5A). To test this directly, we performed a metabolome analysis and monitored the cellular pools of choline and glycine betaine in cells that were grown in the presence or absence of choline (1 mM) and either at low or high (1 M NaCl) osmolarity. Cells grown at low osmolarity did not accumulate choline nor contained glycine betaine. In contrast, those cells that were cultured at high salinity had high glycine betaine pools and possessed moderate intracellular levels of choline, provided that choline had been present in the growth medium. Glycine betaine production by *B. licheniformis* was thus dependent on both the presence of the precursor molecule choline and high-salinity growth conditions (Figure 5C and D), cues that also determine glycine betaine synthesis in *B. subtilis* [59] . 

### Synthesis of proline by *B. licheniformis* for anabolic purposes

Both *B. subtilis* and *B. licheniformis* DSM 13^T^ produce strongly elevated levels of proline when they are osmotically stressed (Figure 1B) [33,34]; the genetics and the enzymes involved in this process have been intensively studied in *B. subtilis* [54,72-74]. As in many other bacteria [75], proline biosynthesis in *B. subtilis* [54,72,73] proceeds from the precursor glutamate [73]. It was therefore somewhat surprising that the synthesis of the GltA protein, a subunit of the glutamate synthase converting 2-oxoglutarate to glutamate, was down-regulated in salt-stressed *B. licheniformis* DSM 13^T^ cells (Figure 3) (Table S2). The same phenomenon has also been observed in *B. subtilis* [41]. 

The two proline biosynthetic routes operating in *B. subtilis* are the ProB-ProA-ProI [72] and the ProJ-ProA-ProH [54] pathways. These pathways serve two different physiological functions, anabolism and osmostress adjustment. The transcriptional control of the involved genes reflects the task to provide the cell with different pools sizes of proline under different growth conditions [53,54,72]. The expression of the genes (*proBA*; *proI*) for the anabolic proline biosynthetic route (ProB-ProA-ProI) are regulated by a *cis*-acting mRNA device, a T-box system [76], that allows enhanced gene expression only when the cells are actually starving for proline [72]. On the other hand, the genes (*proHJ*) for two of the enzymes mediating the osmostress-adaptive proline biosynthetic route ((ProJ-ProA-ProH) are osmotically inducible. These two routes are interlinked in *B. subtilis* by the *proA*-encoded γ-glutamyl-phosphate reductase (ProA). The *proA* gene is part of the T-box regulated proBA operon and is clearly not under osmotic control [72]. 

**Figure 6:**
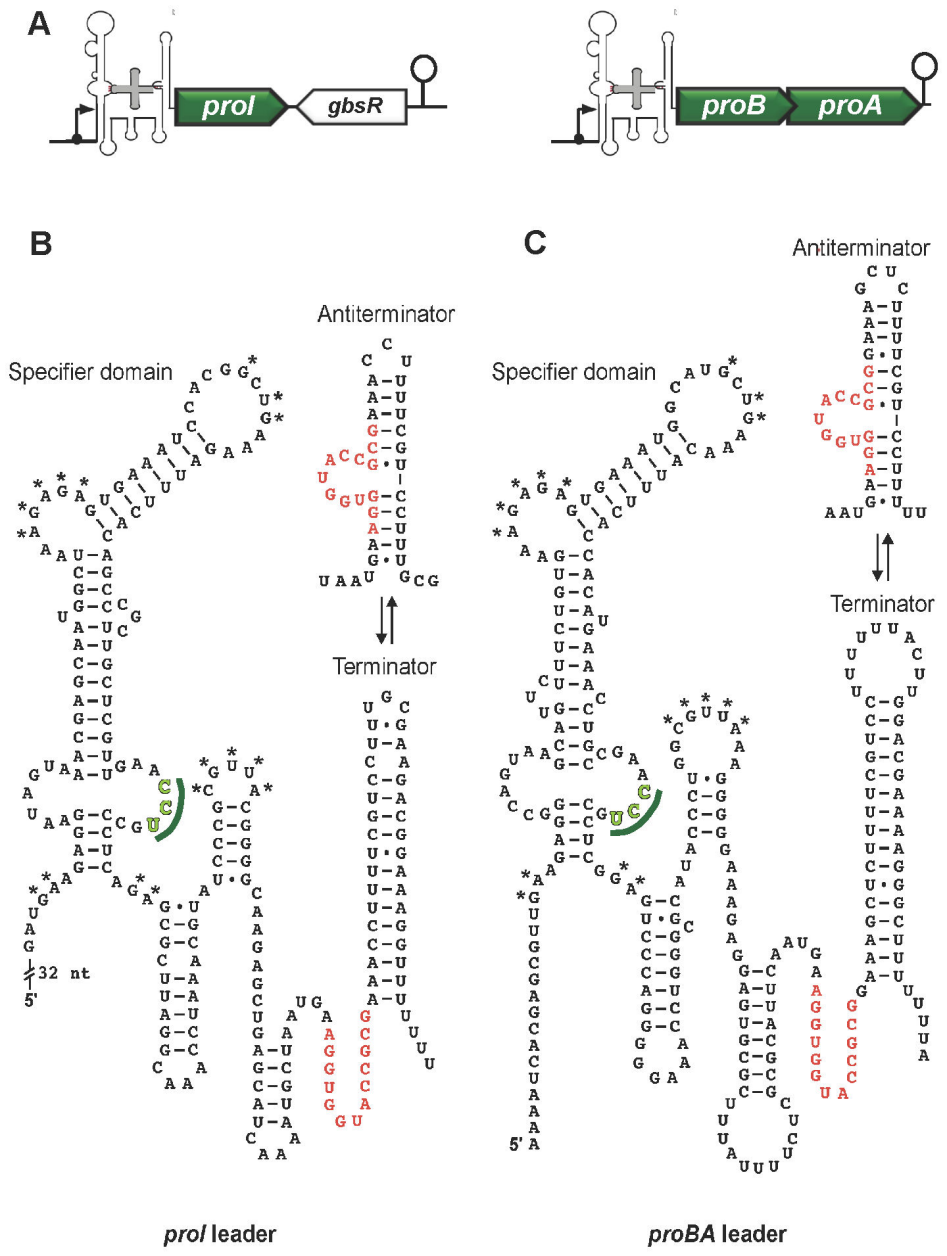
Predicted secondary structures of the *B. licheniformis* DSM 13^T^ *proI* and *proBA* mRNA leader transcripts. (A) Overview of the genetic organization of the structural genes in the genome of *B. licheniformis* DSM 13^T^ [13] for the ProB-ProA-ProI anabolic proline biosynthetic route. The predicted secondary structures of the non-coding 5’-regions of the *proI* (B) and *proBA* (C) mRNA leader sequences were generated with the Mfold algorithm [115] and edited manually for their termination and anti-termination configurations. The suggested proline-specific specifier codons (CCU) [72] in the T-box element for the *proI* and *proBA* mRNA leader sequences are shown in green, and the T-box signature sequences are marked in red. Asterisks indicate other short sequences conserved in the T-box gene family [76,116].

 Mining of the *B. licheniformis* DSM 13^T^ genome [13] revealed the presence of the full set of genes involved in anabolic proline production by *B. subtilis* [72,73] (Figure 4). As outlined above, the transcription of the anabolic *proBA* and *proI* proline biosynthetic genes are controlled via a T-Box system [72], a regulatory device that relies on different conformations of the untranslated 5’-region (UTR) of the mRNA [76,77]. Folding of the 5’-UTR into mutually exclusive terminator and anti-terminator structures is governed by the relative amounts of a given set of charged and non-charged tRNAs. Specificity to a given T-Box system is conferred by a codon, the so-called specifier, present in a bulged region of the folded 5’-UTR of the mRNA with which a cognate tRNA can interact [76-78]. We inspected the UTR regions in front of the *proBA* and *proI* genes for DNA signature sequences that are characteristic for T-box controlled genes [76] and readily detected them (Figure 6A). In Figure 6B and C we present the predicted terminator and anti-terminator configurations of the 5’-UTR for the *proBA* and *proI* mRNA species, respectively. Both the *proBA* and the *proI* sequences contain a proline-specific codon (CCU) in a region of the folded mRNA where the specifier codon is typically located (Figure 6B and C) [76]. We thus surmise that the anabolic proline biosynthetic route (ProB-ProA-ProI) of *B. licheniformis* DSM 13^T^ is genetically controlled by a T-Box system. Like *B. subtilis* [73], *B. licheniformis* DSM 13^T^ possesses genes for multiple Δ^1^-pyrroline-5-carboxylate reductases; one of them is ProG (Figure 4). However, in contrast to the *proBA* and *proI* genes, *proG* does not possess the signature sequence of a T-Box system. The precise physiological function of ProG in both *B. subtilis* and in *B. licheniformis* DSM 13^T^ is unresolved; expression of *proG* was not osmotically inducible under the conditions tested (Figure 2A).

### Osmostress-adaptive proline biosynthetic route

 The genetics of the regulatory switch of the T-Box system controlling the expression of the anabolic *proHJ* and *proI* genes is set such that a wasteful overproduction of proline is prevented through the coupling of the size of the cellular proline pool to the ongoing protein biosynthetic activities of the cell [72]. It is obvious from this type of genetic control that the ProB-ProA-ProI proline biosynthetic route (Figure 4) is unsuited for the biosynthesis of the very large amounts of proline required to achieve osmostress protection (Figure 2A) [53,54,57,58]. Mining of the *B. licheniformis* DSM 13^T^ genome [13] revealed an important difference between *B. subtilis* and *B. licheniformis* concerning the genes for the osmostress-adaptive proline biosynthetic route, since a gene cluster (*proH-proJ-proAA*) was detected that encodes the full complement of enzymes required for proline biosynthesis (Figure 4). This latter group of genes, in contrast to those involved in the anabolic proline production (ProB-ProA-ProI), was found to be osmotically inducible in our DNA transcriptional profiling study (Figure 2A).

**Figure 7:**
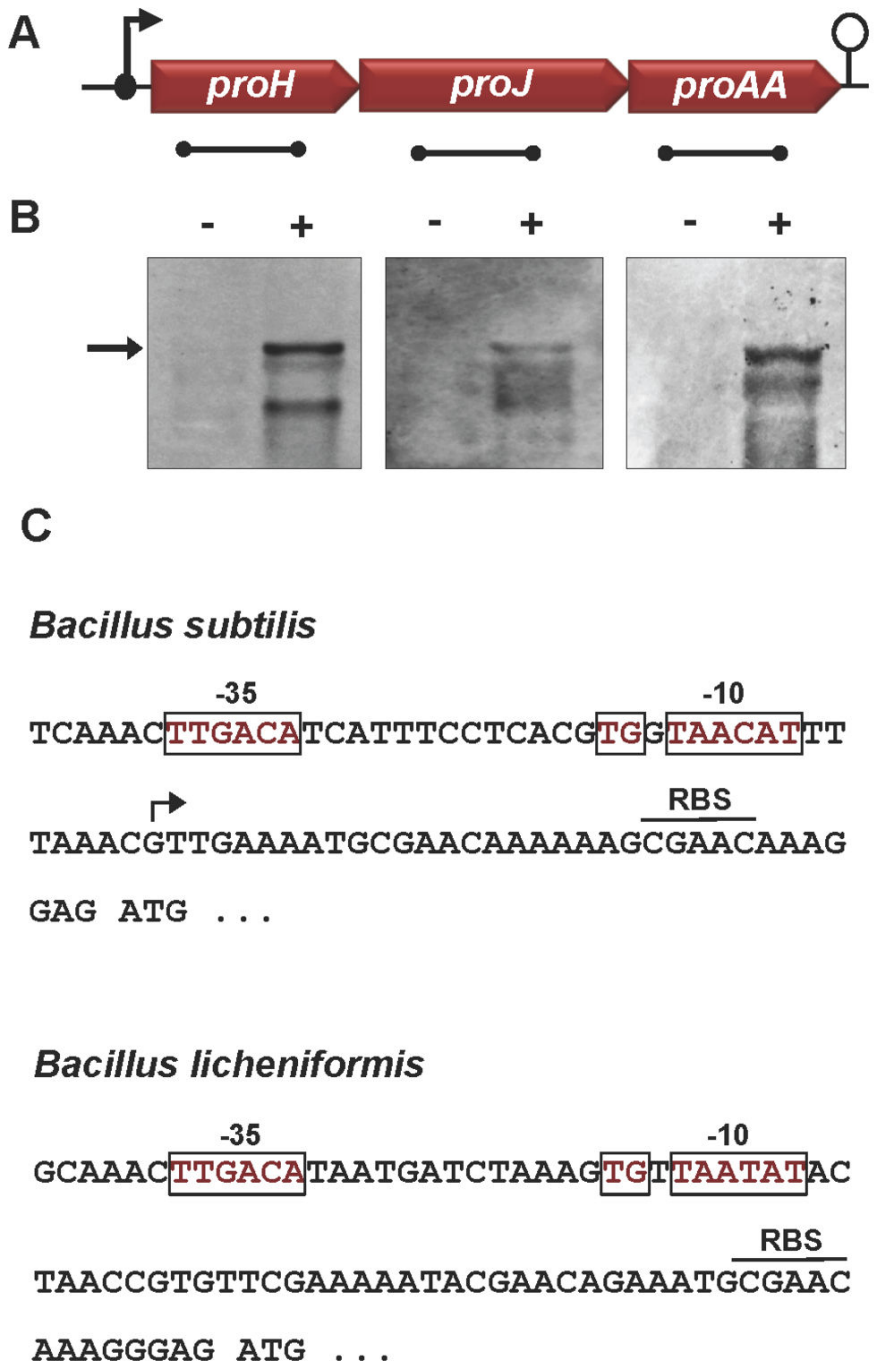
Induction of *proHJAA* transcription in *B. licheniformis* DSM 13^T^ in response to salt stress. (A) Genetic organization of the *B. licheniformis* DSM 13^T^ *proHJAA* locus with its indicated promoter and transcriptional terminator regions. The localization of the single-stranded anti-sense RNA used as probes in the Northern blot analysis of the *proHJAA* gene cluster are indicated as black bars below the individual gene symbols. (B) Northern blot analysis of the *proHJAA* transcript. Total RNA was isolated from cultures of *B. licheniformis* DSM 13^T^ that were grown in SMM either in the absence (-) or the presence (+) of 0.8 M NaCl. Gene-specific RNA transcripts were identified by hybridization of total RNA to DIG-labeled single-stranded anti-sense RNA probes. The arrow indicates the position of an approximately 3,400 nucleotide mRNA species that corresponds to the full-length mRNA of the *proHJAA* operon. (C) DNA sequence of the *proH* promoter regions of the *B. subtilis* and *B. licheniformis* DSM 13^T^ chromosomes. The start site (indicated by an arrow) mapped for the *B. subtilis* *proHJ* mRNA transcript via primer extension analysis [54] revealed a SigA–type promoter (shown in red, with boxed -10, -16 and -35 sequences [79]) and a putative ribosome-binding site (RBS) located upstream of the predicted ATG start codon of the *proH* coding region. DNA sequences resembling those of the *B. subtilis* *proHJ* promoter [54] can be found in the *B. licheniformis* DSM 13^T^ *proHJAA* promoter region.

**Figure 8:**
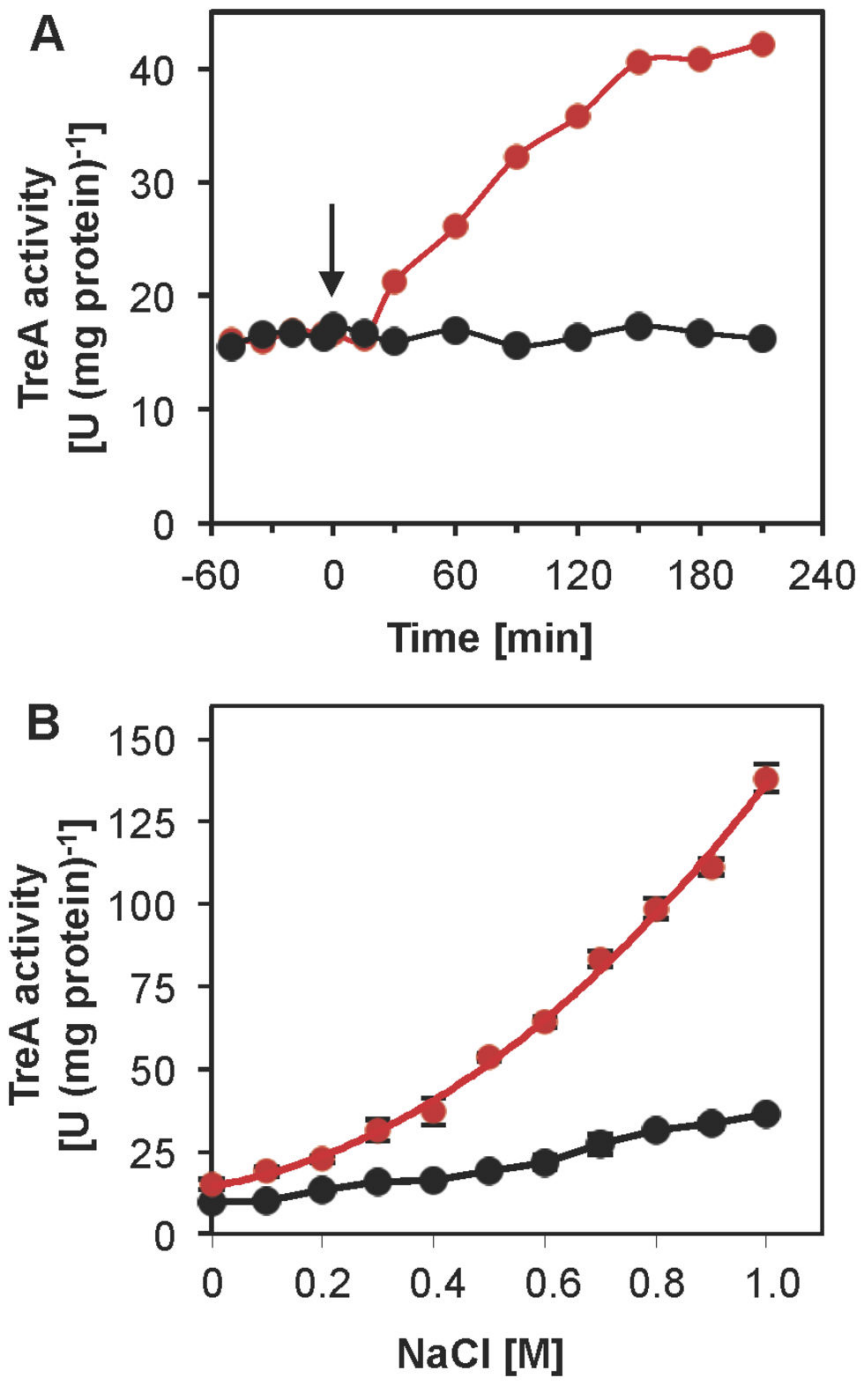
Osmotic induction of *proHJAA* promoter activity in response to an osmotic up-shock and under continuous stress conditions. A 130-base pair DNA fragment covering the predicted promoter of the *proHJAA* operon from *B. licheniformis* DSM 13^T^ was fused to a promoterless *treA* reporter gene [Φ(*proHB.li 130´-treA*) *cat*] and inserted into the chromosome of the heterologous *B. subtilis* host strain FSB1 [(*treA::neo*)1] [43], thereby yielding the reporter strain MDB60. (A) The expression of the [Φ(*proH_130_-treA*) *cat*] reporter fusion was monitored by measuring the TreA activity of strain MDB60 cells cultivated in SMM that were subjected either to a sudden osmotic up-shock (indicated by an arrow) with 0.4 M NaCl (red symbol) or that were not subjected to an osmotic up-shift (black symbol). (B) Cultures of the Φ(*proH_130_-treA*) reporter fusion strain MDB60 were grown in SMM with different salinities either in the absence (red symbol) or in the presence of 1 mM of the osmoprotectant glycine betaine (black symbol). They were assayed for TreA reporter enzyme activity when the *B. subtilis* cells reached mid-exponential growth phase (OD_578_ of about 1.5). The values given for the TreA enzyme activity represent the averages of two independently grown cultures. For each sample analyzed, the TreA activity was determined twice.

A Northern blot analysis revealed that the *proH*, *proJ*, and *proAA* genes are co-transcribed as an osmotically inducible operon (Figure 7A and B). Inspection of the DNA sequence in the presumed regulatory region of the *B. licheniformis* DSM 13^T^ *proH-proJ-proAA* gene cluster revealed a putative SigA-dependent promoter (Figure 7C), whose -10, -16 and -35 regions [79] closely resemble those identified for the osmotically inducible *B. subtilis* *proHJ* operon through primer-extension analysis [54]. To study the osmotic control of the *proH-proJ-proAA* operon in greater detail, we fused a 130-bp DNA segment encompassing the promoter region to a promoterless *treA* reporter gene and then inserted the resulting *proH-treA* transcriptional fusion in a single copy at the non-essential *amyE* site of the *B. subtilis* genome. A sudden exposure of the *proH-treA* reporter strain to increased salinity (0.4 M NaCl) led to a strong induction of gene expression (Figure 8A). Under sustained high-salinity growth conditions, the level of transcription of the *proH-treA* reporter fusions was sensitively linked to the severity of the imposed osmotic stress (Figure 8B). Hence, osmotic control of *proH-proJ-proAA* operon transcription was maintained across the species boundaries of *B. licheniformis* DSM 13^T^ and *B. subtilis*. 

**Figure 9:**
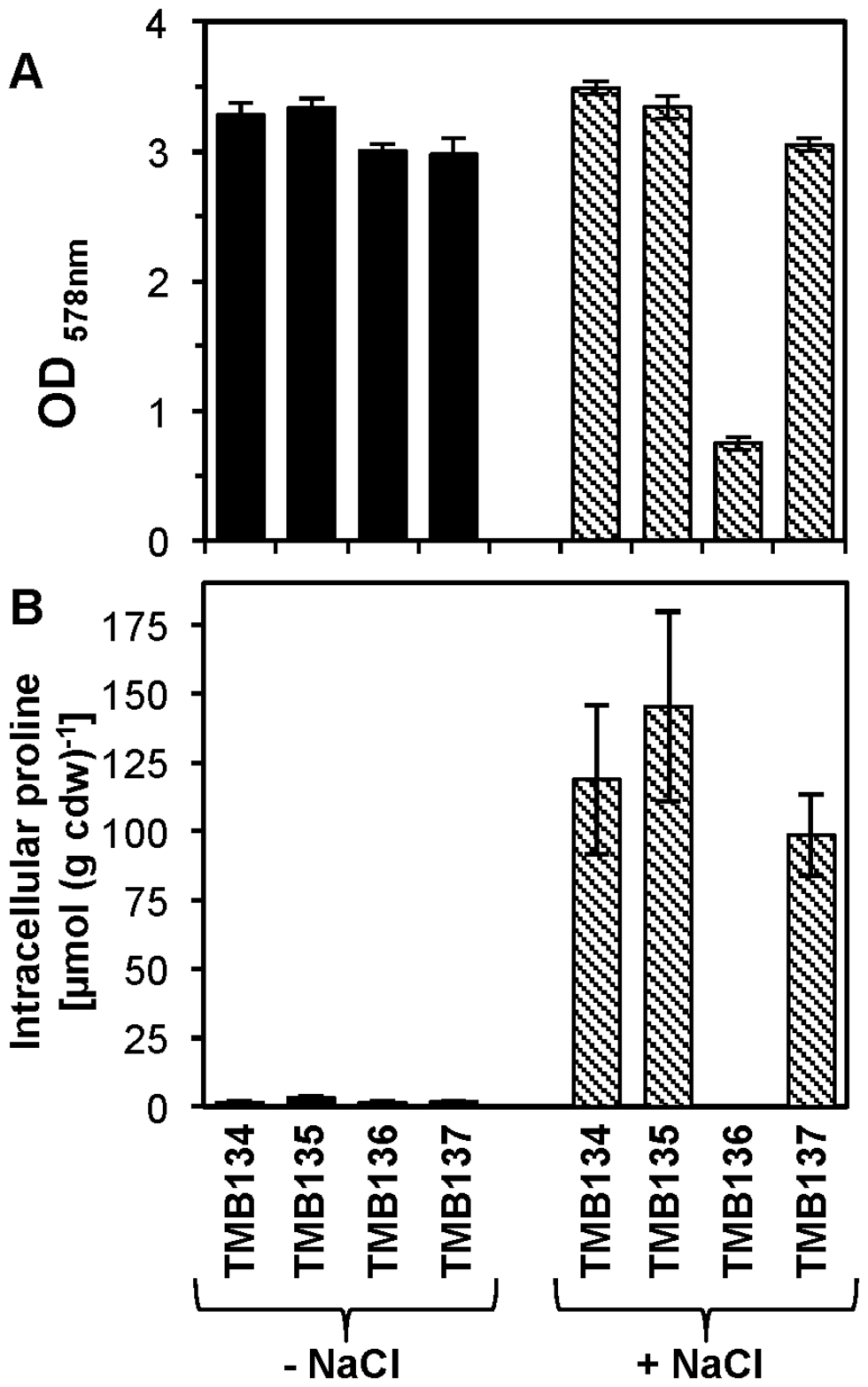
Physiological complementation of a *B. subtilis* *proHJ* mutant strain by the heterologous *proHJAA* operon of *B. licheniformis* DSM 13^T^. The *proHJAA* operon of *B. licheniformis* DSM 13^T^ was cloned into plasmid pX, yielding plasmid pTMB20. pTMB20 and the empty cloning vector pX (used as a control) were recombined in a single copy into the chromosomal *amyE* sites of the *B. subtilis* wild-type strain JH642 and its [Δ(*proHJ*::*tet*)1] mutant derivative JSB8 [72]. This resulted in the construction of the following *B. subtilis* strains: TMB134 [*proHJ* wild type and *amyE*::pX], TMB135 [*proHJ* wild type and *amyE::proHJAA*], TMB136 [Δ(*proHJ*::*tet*)1 and *amyE*::pX], and TMB137 [Δ(*proHJ*::*tet*)1 and *amyE::proHJAA*]. (A) Cultures of these strains were grown in SMM without (black bars) or with 0.8 M NaCl (hatched bars). Their growth yields (OD_578_) were measured after 16 h of incubation at 37 °C. (B) Proline content of recombinant *B. subtilis* strains grown in SMM without (black bars) or with 0.8 M NaCl (hatched bars). When the cultures had reached mid-exponential growth phase (OD_578_ of about 2), the cells were harvested, their total solute pool was extracted and the intracellular proline concentrations were determined by HPLC analysis. The error bars represent the standard deviations of the proline pools found in three independently grown cultures. The same set of strains as that shown in panel (A) was used for this experiment.

To further characterize the functionality of the *proH-proJ-proAA*-encoded enzymes from *B. licheniformis* DSM 13^T^ within the physiological framework of osmostress adaptation, we cloned this gene cluster into a plasmid vector (pX) [80] that allows the integration of cloned genes as single copies into the *amyE* site of the *B. subtilis* genome. In this way we inserted the *B. licheniformis* DSM 13^T^ *proH-proJ-proAA* operon into a *B. subtilis* strain that was proficient in osmostress adaptive proline biosynthesis and one that was defective in this stress response. We then tested both the growth of the resulting recombinant strains under high-salinity conditions and measured their proline pools. As summarized in Figure 9, the *B. licheniformis* DSM 13^T^ *proH-proJ-proAA* gene cluster rescued the salt-sensitive growth phenotype [54] of a *B. subtilis* *proHJ* mutant strain (Figure 9A) and restored osmostress responsive proline production (Figure 9B). Hence, there can be no doubt the *proH-proJ-proAA*-encoded enzymes (Figure 4) are responsible for the osmostress adaptive proline biosynthesis (Figure 1B) of *B. licheniformis* DSM 13^T^. 

An *in silico* analysis of the genome sequence of the industrially relevant species *Bacillus megaterium* has previously suggested the existence of two complete proline biosynthetic production routes (Figure 4) with distinctly different physiological functions [81]. However, the transcriptomic (Figure 2A) and physiological data (Figure 9) reported here for *B. licheniformis* DSM 13^T^ provide now for the first time direct experimental evidence for this notion. Hence, in both *B. licheniformis* DSM 13^T^ and in *B. megaterium*, the curious interconnection observed in *B. subtilis* between the anabolic and osmostress-relieving proline biosynthetic routes via a single ProA enzyme [54,72] is avoided. 

### Use of proline as a nutrient by *B. licheniformis* DSM 13^T^

**Figure 10:**
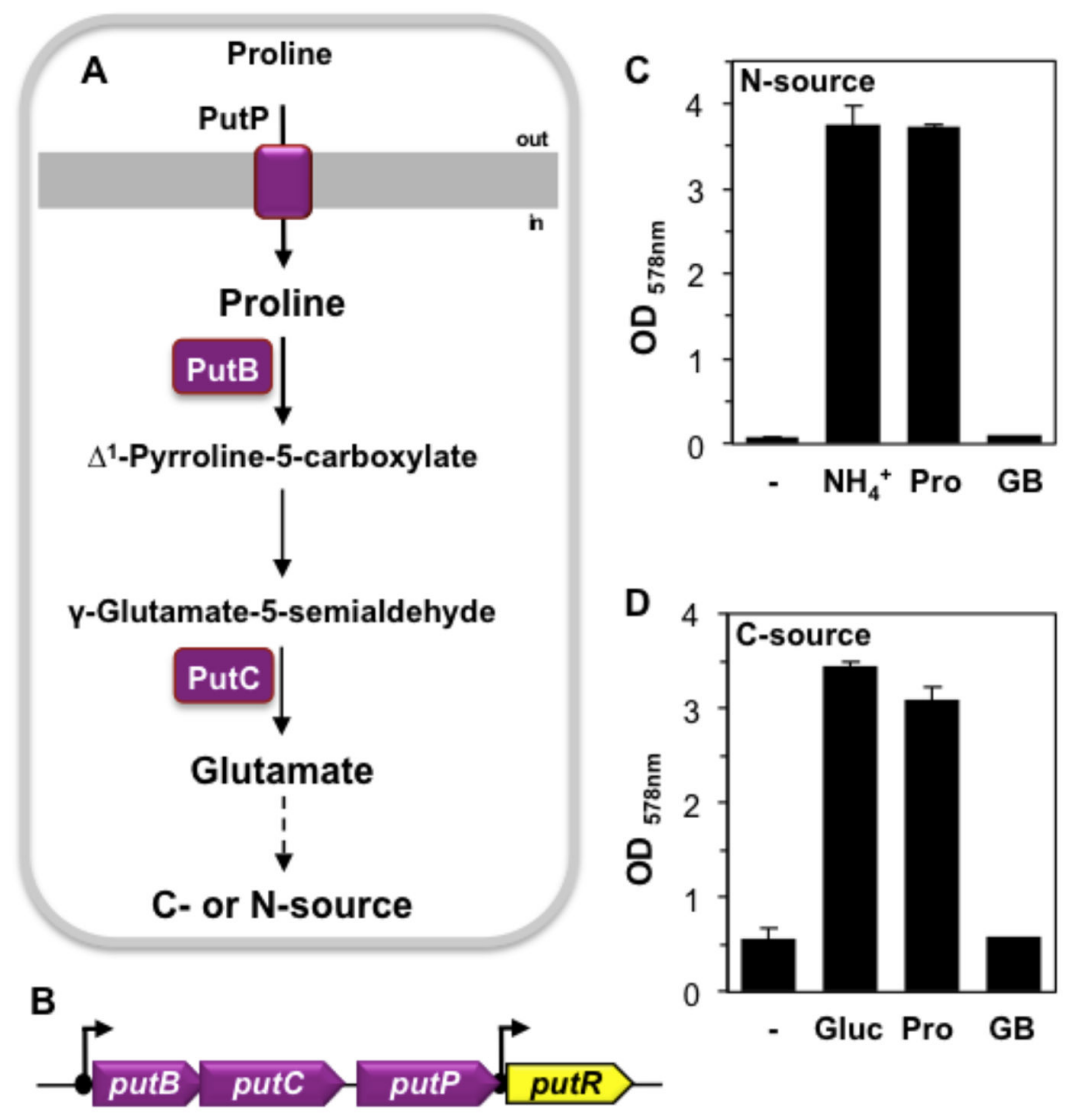
Use of L-proline as a nutrient by *B. licheniformis* DSM 13^T^. The proline catabolic system has been studied in *B. subtilis* where a high-affinity proline transporter (PutP), two proline catabolic enzymes (PutB-PutC) [82], and a proline-responsive activator protein (PutR) [83,84] have been functionally studied. (A) This proline catabolic pathway is predicted from the genome sequence to be present in *B. licheniformis* DSM 13^T^ as well [13]. (B) Genetic organization of the *put* locus of *B. licheniformis* DSM 13^T^. (C) *B. licheniformis* DSM 13^T^ cells were grown in SMM with glucose as the carbon source without the addition of a nitrogen (N) source (-) or in the presence of 15 mM (NH_4_)_2_SO_4_ [NH_4_^+^, 30 mM L-proline [Pro], or 30 mM glycine betaine [GB], respectively. Growth yields of the cultures were measured after 16 h of incubation at 37 °C in a shaking water bath. (D) *B. licheniformis* DSM 13^T^ cells were grown in SMM with (NH_4_)_2_SO_4_ [15 mM] in the absence (-) of a carbon source or in the presence of 27 mM glucose [Gluc], 32.4 mM L-proline [Pro] or 32.4 mM glycine betaine [GB]. Growth yields of the cultures were measured after 16 h of incubation at 37 °C in a shaking water bath. The error bars give the standard deviation of three independently grown cultures.

 In *B. subtilis*, proline not only serves as an osmostress protectant [53,54,58] but can also be exploited by the cell as sole source of energy, carbon, or nitrogen [82]. This is accomplished through proline import via the OpuE-related proline transporter PutP and its subsequent catabolism to glutamate through the sequential actions of the PutB and PutC enzymes [82] (Figure 10A). The corresponding gene cluster (*putBCP*) (*ycgMNO*) is also present in the genome sequence of *B. licheniformis* DSM 13^T^ [13] and is followed by the *putR* (*ycgP*) gene (Figure 10A) that encodes the proline-responsive transcriptional activator protein of the *putBCP* operon of *B. subtilis* [83,84]. As found in *B. subtilis* [82], none of these genes is osmotically inducible in *B. licheniformis* (Table S2). We tested the use of proline as nutrient by *B. licheniformis* DSM 13^T^ and found that it can be used both as sole carbon and nitrogen source (Figure 10C and D). This contrasts with our findings concerning the use of the osmoprotectant glycine betaine as a nutrient, a compound that is metabolically inert not only in *B. licheniformis* DSM 13^T^ (Figure 10C and D) but in *B. subtilis* as well [60]. 

### General stress responses and ECF controlled systems

Our transcriptome and proteome analysis showed that salt stress caused a strong induction of the σ^B^-controlled general stress regulon of *B. licheniformis* DSM 13^T^ [37,38] (Figure 2B and Figure 3) (Table S1 and S2). At least 23 putative σ^B^-dependent genes revealed a more than ten-fold higher mRNA level after the osmotic up-shock; hence, σ^B^-responsive genes are among the most highly salt-stress inducible genes of *B. licheniformis* DSM 13^T^. This is fully consistent with studies of *B. subtilis*, where salt stress is also one of the most potent inducers of the SigB-dependent general stress response system [39,40]. Among the σ^B^-controlled genes identified in this study (Figure 2B), one can correlate the predicted function(s) of the encoded proteins with particular types of stress-relieving properties for some of them. These are: *bmrU*, a gene that encodes a multidrug resistance protein; *gsiB*, a gene encoding a putative desiccation resistance protein; and *dps*, a gene encoding a DNA-protecting protein. The strong up-regulation of these and other SigB-regulated genes (Figure 2B) clearly indicates that the induction of the general stress response system is an important aspect of the cells attempt to cope with a suddenly imposed increase in the external salinity. 

The most strongly induced genes 20 min after the salt shock encode a FMN-binding split barrel domain protein (YdaG), a so-called general stress protein (GspA, a putative glycosyl transferase), and a putative organic hydroperoxide resistance protein (OhrB or YkzA) (Figure 2B). However, the contribution of a particular σ^B^-controlled gene in coping with high salinity can be difficult to understand in physiological terms. The same experience was true for *B. subtilis*, where a comprehensive deletion and functional analysis study has been conducted to elucidate the contribution of individual genes of the σ^B^-regulon to the cell’s ability to resist the detrimental effects of strong up-shifts in the external salinity [41,42].

The high-level induction of the SigB-controlled hydroperoxide resistance gene *ohrB* and the catalase genes *katE1* and *katE2* indicate that a secondary oxidative stress is triggered by the sudden increase in the external salinity (Figure 2B) (Table S1 and S2). This assumption is supported by the increased synthesis of further distinct oxidative stress marker proteins, such as KatA (catalase), SodA (superoxide dismutase), and AhpCF (alkyl hydroperoxide reductase) (Figure 3) (Table S3). The concentration of the oxidative stress-specific PerR-regulated DNA-binding protein MrgA was increased more than 20-fold at the protein level after the imposition of the salt stress. It is interesting to note that this protein was detected on the 2D-gels only in the monomeric form in salt-stressed cells, although it occurs in both a monomeric and in multimeric forms in *B. licheniformis* DSM 13^T^ cells subjected to a H_2_O_2_-triggered oxidative stress [22]. 

The *sufCDSUB* operon, which encodes a biogenesis system for an iron-sulfur cluster that is essential for the assembly or repair of Fe/S proteins [85], was significantly induced at the transcriptional and translational level in response to high salinity (Figure 2D and Figure 3). This increased demand for iron-sulfur cluster biogenesis might in part be due to oxidative stress provoked by salt challenges and the damage to Fe/S cluster proteins by reactive oxygen species (ROS). Furthermore, in the proteome of salt-stressed cells a higher concentration of several enzymes of the pentose phosphate pathway could be detected 2 h after the beginning of the salt stress. This could also be related to the onset of a secondary oxidative stress, as many ROS-detoxifying enzymes rely on NADPH [86], which is produced in the pentose phosphate pathway. 

In *B. subtilis* a secondary oxidative stress response is linked to the MgsR transcriptional regulator [87,88]. This σ^B^ dependent regulator seems to sense and integrate the secondary oxidative stress signal and controls a specific subregulon within the σ^B^ dependent general stress regulon. It is interesting to note that the *mgsR* homologue gene *yqgZ* in *B. licheniformis* belongs to the strongest induced genes triggered by salt stress. It has been suggested that members of this MgsR subregulon are linked to oxidative stress management [87,88]. In our study we found an induction of several putative MgsR controlled genes with in most cases unknown functions (e.g. *gsiB*, *ydaD*, *ydaE*, *yhdF*, or *ydbD*). 

The elevated transcription and synthesis of the general stress proteins ClpC, ClpE and ClpP or DnaK, GroEL and GrpE could also indicate damage or misfolding of proteins that might be caused either directly by the osmotic stress or indirectly by the elicited secondary oxidative stress responses (Figure 3) (Tables S1, S2 and S3). Consistent with this idea are reports that show an induction of members of the Clp system in *B. licheniformis* DSM 13^T^ cells directly subjected to oxidative stress [89]. Furthermore, it has already been shown that ClpC is required in *B. subtilis* for resistance to salt stress, as the growth of a *clpC* mutant was significantly impaired under high saline conditions [90]. 

The two sigma factors σ^W^ and σ^X^ with extracytoplasmic function (ECF sigma factors), which govern physiological response to cell envelope stress in *B. subtilis* [91], were affected by salt stress in *B. licheniformis* DSM 13^T^. Among the seven ECF sigma factor regulons present in *B. subtilis* [91], three are affected by a sudden increase in the external osmolarity: σ^M^, σ^W^, and σ^X^ [39,92,93]. While the σ^M^ regulon in *B. subtilis* is known to be crucial for prolonged survival in a high-salt-containing environment due to its central role in cell-wall maintenance systems [93], we did not find any σ^M^-dependent gene induced in *B. licheniformis* after salt stress. In *B. licheniformis* DSM 13^T^ cells, only genes of the two ECF regulons σ^W^ and σ^X^ showed increased induction rates (Figure 2C) (Table S1 and S2). At least 26 ECF-regulated genes of *B. licheniformis* DSM 13^T^ whose transcription was responsive to salt stress could be identified in this study. In addition to the up-regulation of the ECF sigma-factor-encoding genes *sigW* and sig *X*, enzymes involved in cell wall metabolism had higher expression levels at the transcriptional level. This includes, for example, an increased transcription of the intra-membrane protease *yqeZ* and the membrane protein *yvlD*. None of these cell-wall-associated proteins could be identified in the 2D-gel-based proteomic approach employed in this study. The membrane-bound nature of these proteins could readily explain their absence from the soluble cytosolic proteome. However, a higher rate of synthesis and protein level could be detected for the cell-wall-related protein UTP-glucose-1-phosphate uridylyltransferase (GtaB) and the penicillin-binding proteins PbpC and PbpX. Up-regulation of penicillin-binding proteins at the transcriptional level in salt-shocked cells was also observed for *B. subtilis* [39,94]. 

One of the strongest gene clusters of *B. licheniformis* DSM 13^T^ inducible by high salinity (Figure 2A) encode for the components (BLi03671 and BLi03672) of a transport system that belongs to the exporter sub-family of the ABC super-family (Figure 4). Expression of the genes for the membrane component (BLi03672) and ATPase subunit (BLi03671) was about 250- and 136-fold up-regulated in response to high salinity (Table S1 and S2). Homologs of this presumed multi-drug export system do not exist in *B. subtilis* but can be found in the genome sequences of *B. megaterium* [81] and *Bacillus pumilus* [95]. Wecke et al. [20] have shown that the expression of the structural genes for the BLi03671/BLi03672 ABC-type export system from *B. licheniformis* is up-regulated in response to the cell-wall damaging antibiotic vancomycin in a fashion that is independent of ECF-type sigma factors, indicating that its strong salt-stress-mediate induction might reflect cell-wall-damaging effects exerted by severe salt shocks. 

We observed that proteins involved in chemotaxis (CheV, CheW, CheY, Yfms) and motility (MotB, SwrC) were accumulated 120 min subsequent to the imposed salt stress and that the flagellin protein Hag was also present at higher concentrations in salt stressed cells (Table S3). This latter observation conflicts with data from the transcriptome analysis where the *hag* mRNA levels were found to be strongly down-regulated in response to salt stress. However, it needs to be kept in mind that the latest time point taken for the transcriptome analysis was 20 min after the salt stress was imposed, whereas the accumulation of proteins in salt-stressed cells was analyzed 2 h subsequent to the imposition of the salt stress. These findings indicate perhaps a progressive response to osmotic stress as far as the motility and chemotactic systems of *B. licheniformis* are concerned. It should be noted in this context that a sustained rise in the external salinity strongly negatively affects the chemotaxis and motility systems of *B. subtilis* and strongly down-regulate the production of the Hag protein [39,65].

### Comparison of the transcriptomic and proteomic responses

We used a combined transcriptomic and proteomic global gene expression analysis in order to identify relevant genes and gene clusters whose pattern of transcription is altered osmotic stress conditions in *B. licheniformis*. Although the two-dimensional PAGE-based separation of L-[^35^S]-methionine-labeled proteins allows for a good visualization of new highly expressed marker proteins, it shows only a very restricted window of the induced proteins under these stress conditions. Furthermore, as revealed in many other gene expression profiling studies, there is no direct quantitative correlation between the transcript and the protein levels. In most cases induction ratios at the transcriptomic level are significantly higher than at the proteomic level. This was also observed in our study. One typical example is the gene encoding the organic hydroperoxide resistance protein OhrB (YkzA) (see Table S2 and S3), which revealed a remarkable induction ration in osmotic stress that exceeded 500-fold at the transcript level but led to only a maximal 10-fold induction at the proteomic level. The overall expression pattern of the detectable proteins was in most cases consistent with their mRNA levels. There are very few exceptions; as an example, *hag* in this study, we detected a clear down-regulation of the *hag* gene at the transcriptomic level but observed a significantly increased accumulation at the proteomic level.

### Concluding remarks

Our study offers an in-depth analysis of the salt-stress response of *B. licheniformis* at the transcriptome and proteome level, and we have combined this system-wide assessment with physiological approaches that address the synthesis and uptake of osmoprotectants. Combined with genome mining of systems known to be important for the management of osmotic stress in *B. subtilis* [32,41,45,52,54,69,71], the data presented here provide a detailed view of the *B. licheniformis* cell’s attempt to cope with and ameliorate the negative effects of high salinity on its physiology. A considerable overlap with the salt stress response of *B. subtilis* was evident but also stress reactions that are specific to *B. licheniformis* were found. 

It well established that large-scale growth conditions of microbial cells influence the outcome of industrial size bioprocesses [96,97]. High-level excretion of the desired product from the microbial producer cell into the culture broth will successively increase the osmolarity of the medium [30,31] and such an increase can limit cell density and volumetric productivity. The feeding of osmoprotective compounds such as glycine betaine, choline, carnitine, proline or proline-containing peptides to osmotically stressed cells [58,60,71] will likely ameliorate such negative effects (Figure 1C) [30]. 

Microorganisms used in large-scale reactor environments are continuously exposed to various types of gradients [96,98]. This comprises also the development of osmotic gradients as a result of the feeding of high concentrated nutrient solutions and insufficient mixing of the culture broth. Since such osmotic gradients will certainly induce stress response in the microbial cell factory [28,31], the data presented here will help to understand such processes in *B. licheniformis* on a much more solid footing. Furthermore, the ability to distinguish between essential osmotic-stress-relieving pathways and dispensable regulons, which are not required or are even perturbing under industrial scale process conditions, could help to design more robust and efficient production strains of *B. licheniformis*. 

We surmise that the *B. licheniformis* DSM 13^T^-derived *proH-proI-proAA* operon for the osmostress-adaptive proline biosynthesis (Figure 4) might be exploited in the context of synthetic microbiology as a *bio-brick* to engineer salt stress resistance in salt-susceptible microorganisms. Furthermore, the salient features of the osmotically inducible promoter driving the expression of this gene cluster (Figures 7 and 8), and the regulatory elements of other osmotically controlled *B. licheniformis* genes identified in our study (Figure 2) (Table S1), might turn out to be useful tools in developing novel types of environmentally responsive expression systems for Bacilli.

## Materials and Methods

### Bacterial strains, media and growth conditions

The *Escherichia coli* K-12 strain DH5α (Invitrogen, Carlsbad, CA, USA) was used for routine cloning purposes, maintenance of cloning vectors, and recombinant plasmids. These strains were maintained on Luria-Bertani agar plates [99]. For physiological studies that involved either *B. subtilis* or *B. licheniformis*, the cells were cultured in Spizizen´s minimal medium (SMM) [100], with 0.5% (w/v) glucose as the carbon source. In experiments that employed the *B. subtilis* strain JH642 (*trpC2 pheA1*) [101] or any of its derivatives, the minimal medium was supplemented with L-tryptophan (20 mg l^-1^) and L-phenylalanine (18 mg l^-1^) to satisfy the auxotrophic growth requirements of JH642. A solution of trace elements [100] was added to SMM to improve the growth of *B. subtilis* and *B. licheniformis* strains. The growth of bacterial cultures was monitored by measuring their optical densities at a wavelength of 578 nm (OD_578_). In physiological experiments, cultures were inoculated from exponentially growing pre-cultures into pre-warmed minimal media (20-ml culture volumes in 100-Erlenmeyer flasks) to optical densities OD_578nm_ of 0.1, and the cultures were then further propagated at 37°C in a shaking water bath set to 220 rpm. The osmolarity of SMM was increased by adding NaCl to it from a 5 M stock solution to the final concentration indicated in the individual experiments. Solutions of the compatible solutes glycine betaine, choline, proline, proline betaine, carnitine, choline-O-sulfate, dimethylsulfoniopropionate (DMSP), ectoine, and hydroxyectoine were sterilized by filtration and added to the growth media from 100 mM stock solutions (the final concentration in the medium was 1 mM). The antibiotics ampicillin, chloramphenicol, and tetracycline were used in the final concentrations of 100 µg ml^-1^, 5 µg ml^-1^, and 10 µg ml^-1^, respectively.

For all experiments that involved *B. licheniformis*, we used strain DSM 13^T^ (equivalent to strain ATCC 14580), the *B. licheniformis* type strain of the German Collection of Microorganisms and Cell Cultures (DSMZ GmbH, Braunschweig, Germany). Its genome sequence has been established [13]. To perform proteomic and transcriptomic analyses of *B. licheniformis* DSM 13^T^, the cells were cultivated in synthetic Belitzky minimal medium (BMM) [102] with 0.2% (w/v) glucose as the carbon source. Growth of these cultures was monitored by measuring their optical density at 500 nm (OD_500nm_). Cultures of *B. licheniformis* were grown in 500-ml Erlenmeyer flasks in a shaking water bath at 180 rpm and 37°C and were inoculated to a starting OD_500nm_ of 0.04 from overnight cultures. 

Heterologous expression studies involving the *B. licheniformis* *proHJAA* operon were performed in the *B. subtilis* strain JH642 [101] as the host, or its corresponding *proHJ* mutant derivative, strain JSB8 [Δ(*proHJ*::*tet*)1] [54,72]. Strain FSB1 [(*treA::neo*)1] [43] is a derivative of JH642 and was used as a host for the chromosomal integration of the [Φ(*proHB.li 130´-treA*)1*6* *cat*] reporter construct at the *amyE* region. 

The antibiotics ampicillin, chloramphenicol, and tetracycline and the artificial substrate for the TreA enzyme, *para*-nitrophenyl-α-D-glucopyranoside (α-PNPG), were all purchased from Sigma-Aldrich (Steinheim, Germany). 9-fluorenyl-methoxycarbonyl chloride (FMOC) and 1-adamanatanamine (ADAM) for the derivatization of amino acids quantitated by HPLC analysis were obtained from Grom Analytic (Rottenburg-Hailfingen; Germany); sodium 3-trimethylsilyl-[2,2,3,3-D4]-1-propionic acid (TSP) as a standard for NMR analysis were obtained from Aldrich (Deisenhofen, Germany). All compatible solutes used in this study were from laboratory stocks. 

### Construction of bacterial strains and plasmids

A 3,616-bp DNA fragment containing the *B. licheniformis* DSM 13^T^ *proHJAA* operon region was amplified by PCR from chromosomal DNA using DNA primers [forward primer proH-for: 5’-AAAACTAGTCCAAAGGCTGTTGATCTCC-3’; reverse primer proAA-rev: 5’-AAAACTAGTGGTGTCTGACAAACCAGGTG-3’] that contained artificially added *Spe*I restriction sites at their ends. The amplified genomic region contained the *proHJAA* coding region and a DNA segment (314 bp upstream of the *proH* start codon) that is predicted to contain the *proHJAA* promoter region; it was cloned into the *B. subtilis* vector pX (*cat*) [80], and this yielded plasmid pTMB20. pTMB20 carries the *B. licheniformis* *proHJAA* genes in an anti-linear orientation to the P_XylA_ promoter that is present in plasmid pX [80]. DNA of plasmid pTMB20 and the empty pX cloning vector were linearized by restriction digestion and used to transform competent cells of the *B. subtilis* strain JH642 and its [Δ(*proHJ*::*tet*)1] mutant derivative strain JSB8 [72] to stably integrate the cloned *B. licheniformis* *proHJAA* genes into the *B. subtilis* chromosome at the *amyE* gene via a double recombination event. The resulting strains were TMB134 [*proHJ* ^+^ *amyE*::pX-*cat*], TMB135 [*proHJ* ^+^ *amyE*::*proHJAA-cat*], TMB136 [Δ(*proHJ*::*tet*)1*.amyE*::pX-*cat*], and TMB137 [Δ(*proHJ*::*tet*)1*. amyE*::*proHJAA-cat*]. For the construction of a transcriptional *proH-treA* reporter gene fusion, we amplified a chromosomal 130-bp fragment carrying the predicted *B. licheniformis* *proH* promoter region [forward primer: B.li 130 SmaproHJAAtreA 5´- cgccccgggcaaacttgacataatgatctaaagtg-3’; reverse primer: B.li BamproHJAAtreA 5´- cgcggatcctgatcccgcccctataaaagctacg-3’]; the used DNA primers carried artificially introduced *Sma*I and *Bam*HI restriction sites at their ends. The resulting PCR fragment was cut with the restriction enzymes *Sma*I and *Bam*HI and ligated into the low-copy-number *treA* operon fusion vector pJMB1 (M. Jebbar and E. Bremer, unpublished results), thereby yielding plasmid pMD30. The *treA* gene encodes a highly salt-tolerant phospho-α-(1,1)-glucosidase [103] whose enzyme activity can be quantitated using the chromogenic substrate PNPG [104]. DNA of plasmid pMD30 [Φ(*proHB.li 130´-treA*)1*6* *cat*] was linearized by digestion with a restriction enzyme and used in a transformation experiment to insert the reporter gene fusion construct as a single copy into the chromosomal *amyE* locus of the *B. subtilis* strain FSB1 [(*treA::neo*)1] [43] via a double recombination event by selecting for chloramphenicol-resistant colonies; this yielded strain MDB60. Loss of AmyE function caused by the integration of the [Φ(*proHB.li 130´-treA*)1*6* *cat*] construct into *amyE* was assessed by flooding *B. subtilis* colonies that were grown on agar plates containing 1 % starch with Gram´s iodine stain and scoring the size of the zone around individual colonies where starch hydrolysis had occurred [105]. 

### TreA enzyme assay

To determine the transcriptional activity of the *B. licheniformis* *proHJAA* promoter in a heterologous *B. subtilis* host, we grew the [Φ(*proHB.li 130´-treA*)1*6* *cat*] reporter strain MDB60 in SMM with various NaCl concentrations and in the absence or presence of 1 mM glycine betaine. When the cells reached mid-exponential growth phase (OD_578_ of about 1.5), 1.5 ml of each culture was harvested by centrifugation and assayed for TreA reporter enzyme activity using the chromogenic substrate PNPG as described [99,104]. TreA enzyme activity is represented in units per mg of protein, and the protein concentrations of the samples were estimated from the optical density of the cell [99]. 

### HPLC analysis of intracellular proline pools in cells adapted to hyperosmotic stress

For the quantification of the newly synthesized compatible solute proline, *B. subtilis* cultures were grown in SMM in the presence of various NaCl concentrations. After they reached mid-exponential growth phase (OD_578_ of about 2.0), the cells were harvested by centrifugation and lyophilized; the cell dry weight (cdw) was then determined. Soluble compounds were extracted from the dried cells by the Blight and Dyer technique using a methanol-chloroform-water mixture (10:5:4) (vol/vol/vol) [34]. The amino acids present in the extracts were separated as FMOC (9-fluorenyl-methoxycarbonyl chloride)-derivatives in an HPLC analysis and using a GROM-SIL 11 Amino-1PR column (GROM, Herrenberg, Germany) coupled to a fluorescence detector. Quantification of the L-proline concentration of the samples was carried out using a standard curve derived from the parallel measurement of various defined standard solutions of FMOC-derivatized L-proline.

### Northern blot analysis

Total RNAs was isolated from *B. licheniformis* DSM 13^T^ cultures that were grown in SMM either in the absence or in the presence of 0.8 M NaCl to mid-exponential growth phase (OD_578_ of approximately 1.5) by the acidic-phenol method [106]. 15 µg of total RNAs were denatured by heating (70°C, 5 minutes) in the presence of 50 % formamide and separated according to size on a 1.4 % agarose gel. Digoxygenin-labeled single-stranded antisense RNA probes covering internal 500-bp regions of the *proH*, *proJ*, and *proAA* genes were generated using the *in vitro* transcription system StripEZ-kit (Ambion, Austin, Texas, USA). PCR fragments of the above-indicated gene regions carrying an artificial T7 promoter sequence at one of their ends were used as DNA templates for the *in vitro* transcription reaction to generate the single-stranded anti-sense RNA probes. Northern blot analysis of the denatured RNA from *B. licheniformis* DSM 13^T^ was performed as described [107]. 

### Exposure of *B. licheniformis* to salt shock conditions and cell sampling for proteome and transcriptome analysis

Exponentially growing *B. licheniformis* cells (OD_500nm_ of about 0.4) were subjected to a strong salt shock by the addition of NaCl to a final concentration of 1.0 M (6 g of solid NaCl was added per 100 ml of cell culture). Cell samples for RNA extraction were taken from unstressed cultures before (control) and 5, 10, and 20 min after NaCl was added to the cultures. The cells withdrawn from the cultures were mixed with 0.5 volumes of ice-cold killing buffer (20 mM Tris-HCl pH 7.5, 5 mM MgCl, 20 mM NaN_3_), and the cells were then immediately harvested at 10 000 x g for 5 min at 4°C. Labeled cytoplasmic proteins were prepared by incubating the bacteria with 556 Bq ml^-1^ L-[^35^S]-methionine (specific activity 37 TBq/ mmol; Hartmann Analytic GmbH, Braunschweig, Germany) for 5 min, as described [21]. Cells were labeled during exponential growth phase (OD_500nm_ 0.4, control) of the *B. licheniformis* culture, and 10 and 30 min after the addition of NaCl (final concentration 1.0 M). For the identification of proteins, preparative SDS gels with unlabeled proteins were prepared using cells treated with NaCl for 30 and 60 min as described by Hoi et al. [21]. In addition, cell samples were harvested 2 h after the osmotic up-shock (the control was harvested at an OD_500nm_ of 0.4 before the salt stress) for a label-free quantification method by using the Synapt G2 mass spectrometer technique (Waters; Milford, MA; USA). 

### RNA isolation and DNA microarray experiments

To monitor gene expression before and subsequent to a suddenly imposed salt stress, total RNA was isolated from *B. licheniformis* DSM 13^T^ cultures and used for genome-wide transcriptional profiling experiments. The isolation of RNA, the determination of RNA quality and quantity and the microarray experiments were all carried out according to procedures detailed by Schroeter et al. [22]. Custom-made *B. licheniformis* DSM 13^T^ gene expression arrays were prepared by and purchased from Agilent Technologies (Santa Clara, CA; USA) (https://earray.chem.agilent.com/earray/). The design of the hybridization probes was conducted on the basis of the annotated open reading frames of the genome sequence of the *B. licheniformis* DSM 13^T^ strain [13]. Hybridization conditions for the DNA array experiments were as previously described [22]. Microarrays were scanned using the Agilent scanner Type G2565CA combined with the high-resolution upgrade G2539A and the software Scan Control 8.4.1 (Agilent Technologies; Santa Clara, CA; USA). Data were extracted from scanned images using Agilent's Feature Extraction Software (version 10.5.1.1) (Agilent Technologies; Santa Clara, CA; USA) using default settings. Gene expression data were loaded into the Rosetta Resolver® Gene Expression Analysis System 7.2. (Rosetta Inpharmatics c/o Ceiba Solutions; Boston, MA; USA). A common reference type of design was employed, and data from three biological replicate hybridizations were combined using an error-weighted average (Rosetta Resolver error model [108]). Genes showing significant differences in expression were identified by error-weighted ANOVA analysis, with a Benjamini–Hochberg FDR (false discovery rate) multiple test correction. Only genes for which an ANOVA p<0.01 was obtained by statistical testing and whose transcription was at least three-fold induced (fold change above + 3) or three-fold repressed (fold change below -3) for at least one time point throughout the conducted transcriptional profiling experiment were considered as differentially expressed and were used for further evaluation.

#### 2D: PAGE and protein identification

Gel electrophoresis was performed as described previously [109] using 80 µg L-[^35^S]-methionine-labeled protein extract and 500 µg of unlabeled protein extract for preparative gels. In the first dimension IPG-strips (GE Healthcare; Freiburg, Germany) in the pH range 4-7 were applied. Autoradiography of labeled gels and staining of preparative gels was carried out as described by Hoi et al. [21]. Proteins, which were found to be up-regulated in response to salt-stress were cut out from preparative Coomassie-blue-stained gels and identified by mass spectrometry according to Liedert et al. [110]. The identification of proteins down-regulated in response to salt stress was conducted by consulting a *B. licheniformis* proteome reference map established by Voigt et al. [23]. Spot quantification and calculation of synthesis rates of proteins were performed with the Delta 2D software (Decodon GmbH, Greifswald, Germany). The protein-labeling experiments were repeated twice and the synthesis rates (Table S1) were calculated from these two independent experiments. Furthermore, technical replicates were included for the quantification of proteins and their synthesis rates. 

### Sample preparation for label-free protein quantification

Prior to the digestion of proteins with Trypsin, 500 µg of protein was diluted to a final concentration of 5 µg µl^-1^ in 50 mM TEAB/0.1 % RapiGest^TM^ SF buffer (Waters; Milford, MA,; USA). The samples were reduced with 500 µM TCEP (Tris-(2-carboxyethyl)phosphine hydrochloride, Invitrogen) for 45 min at 60°C. The cysteine residues were blocked by alkylation with iodacetamide (Sigma-Aldrich; Steinheim, Germany) for 15 min at room temperature in the dark. Trypsin (Promega; Madison, WI; USA) was added to a final enzyme/protein ratio of 1:20. Subsequently, RapiGest^TM^ was removed from the samples according to manufacturer’s instructions (Waters; Milford, MA; USA). Finally, the protein samples were further cleaned with C18 – StageTips (Proxeon; Odense, Denmark).

### Multiplexed LC/MS analysis (LC/MS^E^)

The digested protein mixture (5 µg) was separated using the nanoACQUITY^TM^ UPLC^TM^ system (Waters; Milford, MA; USA) without a trapping column by direct injection. The protein sample was loaded within a timeframe of 35 min onto the column (nanoACQUITY^TM^ UPLC^TM^ column, BEH130 C18, 1.7 µm, 75 µm x 200 mm, Waters; MA; USA) with 99 % buffer A (0,1% acetic acid in H_2_O_2_), 1 % buffer B (0,1% acetic acid in acetonitrile) at a flow rate of 300 nL/min. The peptides were separated within a 265 min time-frame by applying the following solvent gradient: in 165 min to 18 % buffer B, in 60 min to 26 % buffer B, in 40 min to 60 % buffer B, in 1 min on 99 % buffer B for 10 min, and equilibration for 15 min with 99 % buffer A. The nanoHPLC was coupled online to a Synapt G2 mass spectrometer equipped with a NanoLockSpray source and operated with the MassLynx V4.1 software (Waters; Milford, MA; USA). For all measurements, a mass range of 50-2000 was used and the analyzer was set to resolution mode. For lock mass correction, [Glu1]-fibrinopeptide B solution (GluFib, m/z: 785.8426 Da, ERA, 500 fmol/µL in 50 % (v/v) acetonitrile, 0.1 % (v/v) formic acid) was infused through the reference fluidics system of the Synapt G2 at a constant flow rate of 500 nl/min and sampled every 30 s. The mass spectrometer was run in the LC/MS^E^ mode in which collision energy was alternated between 5eV in the precursor ion trace and a ramp from 10-40 eV for fragment ion trace. All scan times were set to 2 s. All samples were analyzed in triplicate, leading to nine replicate runs per growth state of the *B. licheniformis* culture under study.

### Data evaluation and label-free quantification

For data processing, protein identification and label free quantification, raw data were imported into ProteinLynx Global Server 2.4 (PLGS) (Waters, Milford, MA; USA) and processed via the Apex3D algorithm with the following parameters. Chromatographic peak width: automatic; MS ToF resolution: automatic; lock mass for charge 2: 785.8426 Da/e; lock mass window: 0.25 Da; low energy threshold: 250 cts; elevated energy threshold: 30 cts; retention time window: automatic; intensity threshold of precursor/fragment ion cluster: 1000 cts. Searches for peptides were carried out by the ion accounting algorithm [111] against a randomized protein database predicted from the *B. licheniformis* DSM 13^T^ genome sequence [13] with added common laboratory contaminants (8 478 entries) using the following parameters. Peptide tolerance: automatic; protein tolerance: automatic; minimal fragment ions matches per peptide: 3; minimal fragment ions matches per protein: 7; minimal peptide matches per protein: 2; primary digest reagent: trypsin; missed cleavages: 2; variable modifications: carbamidomethylation C (+57.0215), deamidation N, Q (+0.9840), oxidation M (+15.9949), pyrrolidone carboxylacid N-TERM (-27.9949); false discovery rate (FDR): 5 %; calibration protein: yeast ADH1.

Label-free relative quantification was carried out by Expression^E^ software included in PLGS 2.4 (Waters, Manchester, UK). The quantification was achieved on basis of the observed peptide ion peaks intensities. Normalization was performed on all identified peptide signals using the autonormalization function. Proteins were taken as significantly changed in their amount if regulation probability was below 0.05 or higher than 0.95. To increase the confidence of protein identification and quantification, proteins were filtered out which were identified only once in the nine replicates per growth state of the *B. licheniformis* DSM 13^T^ culture studied. This resulted in a FDR of 3.5 % for the whole dataset used for the analysis.

### Analysis of intracellular metabolites of salt-shocked *B. licheniformis* cells

For the metabolome analysis [112] of salt-stressed *B. licheniformis* DSM 13^T^ cells, cultures growing in BMM without or with 1 mM glycine betaine received a strong salt shock by the addition of NaCl to a final concentration of 1.0 M (6 g of solid NaCl was added per 100 ml of cell culture). Control cultures remained unshocked. In some experiments, the cells were also grown in the presence of 1 mM choline, the precursor for glycine betaine biosynthesis in *B. subtilis* [60,61]. Samples (100 ml at an OD_500_ of about 0.4) of the cell culture were harvested by filtration from unstressed and osmotically stressed cultures as described by Meyer et al. [113]. Cells used for the assessment of intracellular metabolite analysis were washed twice with 2.5 ml isoosmotic saline solution, based on the osmolarity of the unstressed or salt-stressed growth medium. Metabolite extraction was carried out in two steps. Extraction by ethanol was done by alternating shaking and vortexing the filters ten times in the extraction solution (5 ml of 60% (w/v) ethanol, ≤ 4°C) followed by a centrifugation step (5 min, 8500 rpm, 4°C). The supernatant, containing the intracellular metabolites, was transferred into a new Falcon tube. Extraction by water was done by adding 5 ml of ice-cold water to the disrupted filters and the cell pellet, which were then shaken, vortexed, and centrifuged as described above. The aqueous and the ethanolic supernatant from the same sample were combined and were then lyophilized to complete dryness. The identity of intracellular metabolites was analyzed by a modified ^1^H-NMR method as described by Liebeke et al. [114]. Dried samples were resuspended in 500 µl double-distilled water, and 400 µl of each sample were used for subsequent analysis A “noesypresat” pulse sequence with water presaturation and a total number of 1024 free induction decays (FID scans) were used. Spectral referencing and quantification were done relative to 1 mM sodium 3-trimethylsilyl-[2,2,3,3-D4]-1-propionic acid (TSP) in 0.2 M phosphate buffer. 

### In silico analysis of the *B. licheniformis* genome sequence and of the folding of the proBA and proI 5’-UTR mRNA sequences

The mining of the *B. licheniformis* DSM 13^T^ genome [13] for genes involved in osmostress responses were done using the web-server available from the Joint Genomic Institute homepage (http://img.jgi.doe.gov/cgi-bin/w/main.cgi) using the “Find genes-by BLAST” and the “gene neighborhood analysis” tools. The structures of the 5’-untranslated regions (UTR) of the *B. licheniformis* *proI* and *proBA* genes were predicted using the Mfold algorithm [115]. The mRNA secondary structures suggested by the program (http://mfold.rna.albany.edu/) corresponding to the T-box control element [76] were then further manually adjusted based on phylogenetic considerations of other T-box regulated genes [72,116]. 

## Supporting Information

Table S1 **Overview of transcriptionally induced and repressed genes in response to salt stress.**Genes are shown that were significantly induced or repressed at the mRNA level (with a cutoff of +3 or -3, respectively) and for which an ANOVA of p<0.01 was obtained by statistical testing. The information listed in the column ‘‘gene product function’’ was compiled according to functional annotations given by NCBI GenBank AE017333.1 (http://www.ncbi.nlm.nih.gov/nuccore/AE017333) and/or SwissProt (http://www.uniprot.org/uniprot/?query=bacillus+licheniformis&sort=score).
(XLSX)

Table S2 **Summary of all transcriptome data.** For a genome-wide transcriptome analyses of *B. licheniformis* DSM 10^T^, values for each gene and analyzed time point subsequent to the imposition of the salt stress were calculated by the Rosetta Resolver software from three independent array hybridizations against a reference sample taken just before the salt shock (0 min). All values were calculated by the Rosetta Resolvers Gene Expression Analysis System 7.2. (Rosetta Inpharmatics c/o Ceiba Solutions).
(XLSX)

Table S3 **Proteome data for 2D gel analysis (10 and 30 min after addition of NaCl).**
(XLSX)

Table S4 **Proteome data for label free quantification (2 h after addition of NaCl).**
(XLSX)
